# Deciphering the Immune Microenvironment on A Single Archival Formalin-Fixed Paraffin-Embedded Tissue Section by An Immediately Implementable Multiplex Fluorescence Immunostaining Protocol

**DOI:** 10.3390/cancers12092449

**Published:** 2020-08-28

**Authors:** Adrien Guillot, Marlene S. Kohlhepp, Alix Bruneau, Felix Heymann, Frank Tacke

**Affiliations:** Department of Hepatology & Gastroenterology, Charité Universitätsmedizin Berlin, 13353 Berlin, Germany; adrien.guillot@charite.de (A.G.); marlene.kohlhepp@charite.de (M.S.K.); alix.bruneau@charite.de (A.B.); felix.heymann@charite.de (F.H.)

**Keywords:** multiplexing immunohistochemistry, image analysis, immune profiling, liver inflammation, hepatocellular carcinoma, macrophages, fibrosis, steatohepatitis, tumor microenvironment

## Abstract

**Simple Summary:**

Technological breakthroughs have fundamentally changed our understanding on the complexity of tissue organization, both in healthy and diseased conditions. Characterizing the immune cell composition in relation to spatial distribution and histological changes may provide important diagnostic and therapeutic information. Immunohistochemistry remains a method of choice for these purposes, with crucial implications in clinics or in medical research. Nowadays, the widespread use of fluorophore-conjugated antibodies enables simultaneous visualization of an increasing number of proteins on a single tissue slice, with unprecedented resolution. However, advanced methods usually require modern and specific equipment, a significant amount of time for assay optimization, and highly specialized skills. This work reports on the use of a multiplex immunostaining method based on sequential immunostaining and antibody stripping, combined with digital image processing and analysis. Our aim is to provide the medical and research communities with a simple, cost-effective workflow to encompass some current limitations of multiplex immunohistochemistry.

**Abstract:**

Technological breakthroughs have fundamentally changed our understanding on the complexity of the tumor microenvironment at the single-cell level. Characterizing the immune cell composition in relation to spatial distribution and histological changes may provide important diagnostic and therapeutic information. Immunostaining on formalin-fixed paraffin-embedded (FFPE) tissue samples represents a widespread and simple procedure, allowing the visualization of cellular distribution and processes, on preserved tissue structure. Recent advances in microscopy and molecular biology have made multiplexing accessible, yet technically challenging. We herein describe a novel, simple and cost-effective method for a reproducible and highly flexible multiplex immunostaining on archived FFPE tissue samples, which we optimized for solid organs (e.g., liver, intestine, lung, kidney) from mice and humans. Our protocol requires limited specific equipment and reagents, making multiplexing (>12 antibodies) immediately implementable to any histology laboratory routinely performing immunostaining. Using this method on single sections and combining it with automated whole-slide image analysis, we characterize the hepatic immune microenvironment in preclinical mouse models of liver fibrosis, steatohepatitis and hepatocellular carcinoma (HCC) and on human-patient samples with chronic liver diseases. The data provide useful insights into tissue organization and immune–parenchymal cell-to-cell interactions. It also highlights the profound macrophage heterogeneity in liver across premalignant conditions and HCC.

## 1. Introduction

Technological breakthroughs fundamentally have changed our understanding on cellular organization and composition of tissues. Single-cell RNA sequencing, combined with epigenetic and genomic transcriptomics, have revealed a fascinating heterogeneity of parenchymal and non-parenchymal cells in solid organs. The combination of such large data sets with spatial information (“spatial transcriptomics”) and/or cross-validation with proteomic approaches have helped us gain a highly detailed view of zonation, cellular heterogeneity and key distinctive features of the cellular components in tissues such as the liver [1]. These approaches are particularly valuable to understand the nature and composition of inflammatory foci, as immune cells closely interact with parenchymal cells in homeostasis and disease by dynamically adapting their phenotype [2]. Dissecting the immune-cell compartment at high resolution in clinical cohorts and model systems is anticipated to pave the way to novel, tailored interventions for inflammatory and malignant diseases [3,4]. This is of particular relevance for the tumor (immune) microenvironment, because it is currently challenging to predict the individual patient response to novel immune-targeting cancer therapies such as immune checkpoint inhibition [4]. However, for clinical translation, large numbers of samples from preclinical (rodent) models and patient cohorts—as well as large tissue areas—need to be screened, for which “omics” approaches may be of limited value due to technical (e.g., analysis based on small number of cells or tissue) or financial restrictions. To date, immunohistochemistry with a small set of markers from paraffin-embedded tissue sections is considered the standard method for phenotyping immune-cell composition and polarization.

Immunohistochemistry using primary antibodies directed against the antigen of interest on tissue sections dates back a century [5]. Since this time, the technique rapidly gained importance, notably for diagnostic and research purposes, as it provides information in addition to the mere structure of the tissue. Most commonly, a primary antibody is used in combination with an enzymatic reaction (e.g., horseradish peroxidase, HRP or alkaline phosphatase, AP) to provide crucial information on cell distribution or disease stage, for instance. This is classically combined with chemical reactions such as hematoxylin/eosin or Sirius red counterstaining. Over the past years, multiplexing on formalin-fixed paraffin-embedded (FFPE) tissue has become possible, through the simultaneous use of HRP and AP conjugated secondary antibodies or alternative approaches that include sequential staining and chromogen removal [6,7,8]. Another approach consists of using serial sections and comparing similar areas on distinctively stained sections. However, most advances in multiplexing have been achieved by using fluorochrome-conjugated primary or secondary antibodies. Currently, it is possible to stain a certain number of antigens simultaneously, depending on the microscope setup. Alternatively, several commercial kits allow for multiplex immunostaining, but these methods are limited in terms of flexibility and number of targets that can be stained simultaneously on the same section. Overall, major drawbacks of currently available multiplex techniques include their limited flexibility, high reagent costs and the necessity to have access to special equipment.

We herein propose a comprehensive and simple workflow based on sequential immunostaining and antibody stripping, which can be rapidly implemented in any laboratory as a routine method with few adjustments and moderate costs. The method herein described recapitulates the benefits of classical immunohistochemistry, meaning a clear visualization of tissue structures thanks to the use of FFPE samples and takes advantage of the antibody library validated for diagnostic purposes or each laboratory’s most used reagents. The proposed protocol is intended to gain an enormous amount of information from a single sample using multiplex immunostaining and (partially automated) image analysis. Importantly, our method neither requires chemical modifications of the primary antibodies, nor particular tissue preparation. Any existing archival FFPE sample may be used. We have implemented this methodology to allow the in-depth phenotyping of macrophage subsets and other immune cell populations in mouse and human liver as well as the unbiased analysis of large-field scans from a single section, but we show that the adaptable approach can be easily applied to other tissues and research questions as well [9].

## 2. Results

### 2.1. In-Depth Tissue and Cell Phenotyping with Multiplex Immunostaining of Archival Mouse and Human Liver

**Figure 1:**
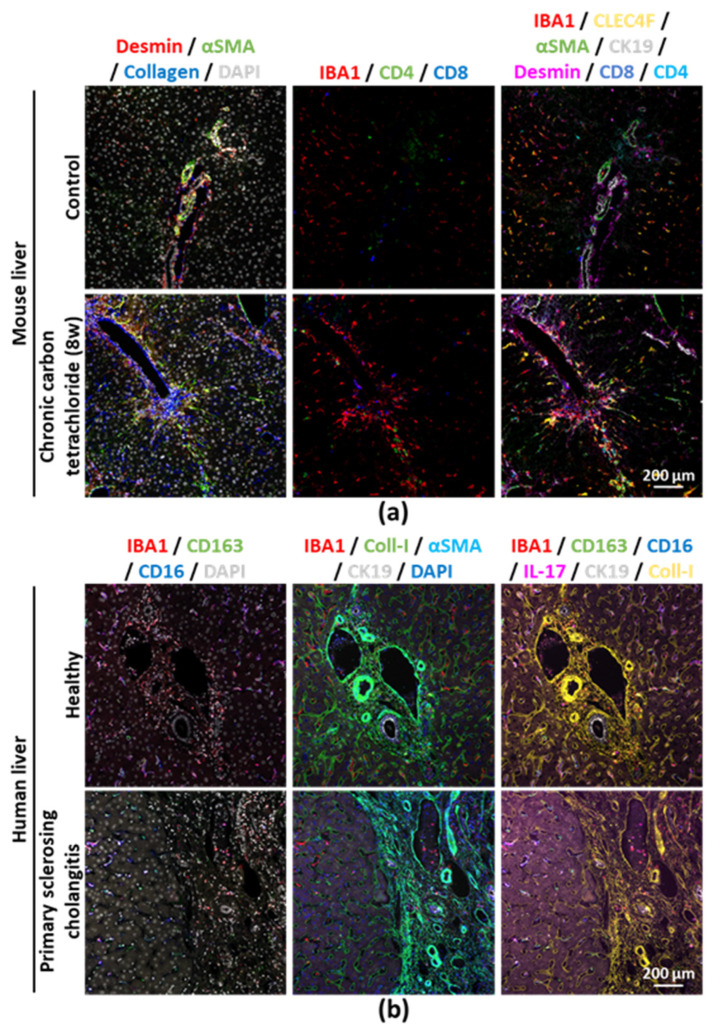
(**a**) Formalin-fixed paraffin-embedded (FFPE) tissue sections from (**a**) healthy and fibrotic mouse liver (chronically injected with carbon tetrachloride (CCl_4_) for 8 weeks) or (**b**) healthy and chronically injured (primary sclerosing cholangitis) human liver were subjected to the multiplex immunostaining method. Single channel pictures provided in Figures S3a,b, and S4a,b.

In order to provide detailed immune phenotyping of mouse and human liver sections, we have established a typical workflow for FFPE tissue processing, immunostaining and image analysis (Table 1 and Figure S1). We exemplarily applied this method to a mouse liver section that had undergone five cycles of staining. We then merged the five DAPI images (Figure S2). Importantly, we were able to take successive pictures of the same area, process the pictures and align them afterwards, while the tissue did not suffer from any structural alteration on the course of the protocol. Accordingly, this also demonstrated that DAPI may be used as a reference for different sets of pictures to be aligned and to reveal potential tissue damage. We then processed the additional images acquired simultaneously after immunostaining, using unconjugated primary antibodies directed against the antigens of interest (Figure 1a,b and Figure S3a,b). Importantly, these results confirmed that the stripping method allowed for the use of primary antibodies generated in the same hosts over two consecutive days, without residual signal from the previous staining (e.g., CLEC4F/CK19/CD4/CD8 and IBA1/desmin, which are all rat- or rabbit-derived antibodies, respectively) (Figure 1a). This allows to distinguish resident hepatic macrophages, i.e., Kupffer cells (IBA1^+^CLEC4F^+^), from monocyte-derived macrophages (IBA1^+^CLEC4F^−^) and their specific localization in proximity to other immune (e.g., CD4^+^ or CD8^+^ T-cells) and hepatic cells (e.g., CK19^+^ cholangiocytes or αSMA^+^Desmin^+^ stellate cells) [10,11]. At the end of the fluorescence image acquisitions, we performed a Masson’s trichrome staining and successfully aligned this bright field and the fluorescence pictures (Figure S3a,b). Importantly, we used the same approach for multiplex immunostaining on human liver sections (such as primary sclerosing cholangitis, a premalignant condition for hepatobiliary tumors), demonstrating that this method is applicable to available archival clinical samples from patients, provided that interspecies marker discrepancies are taken into account in the panel design, when applicable (Figure 1b and Figure S4a,b).

### 2.2. Generating Detailed Spatial and Phenotypic Information from Large Area Scanning on Different Organs

**Figure 2:**
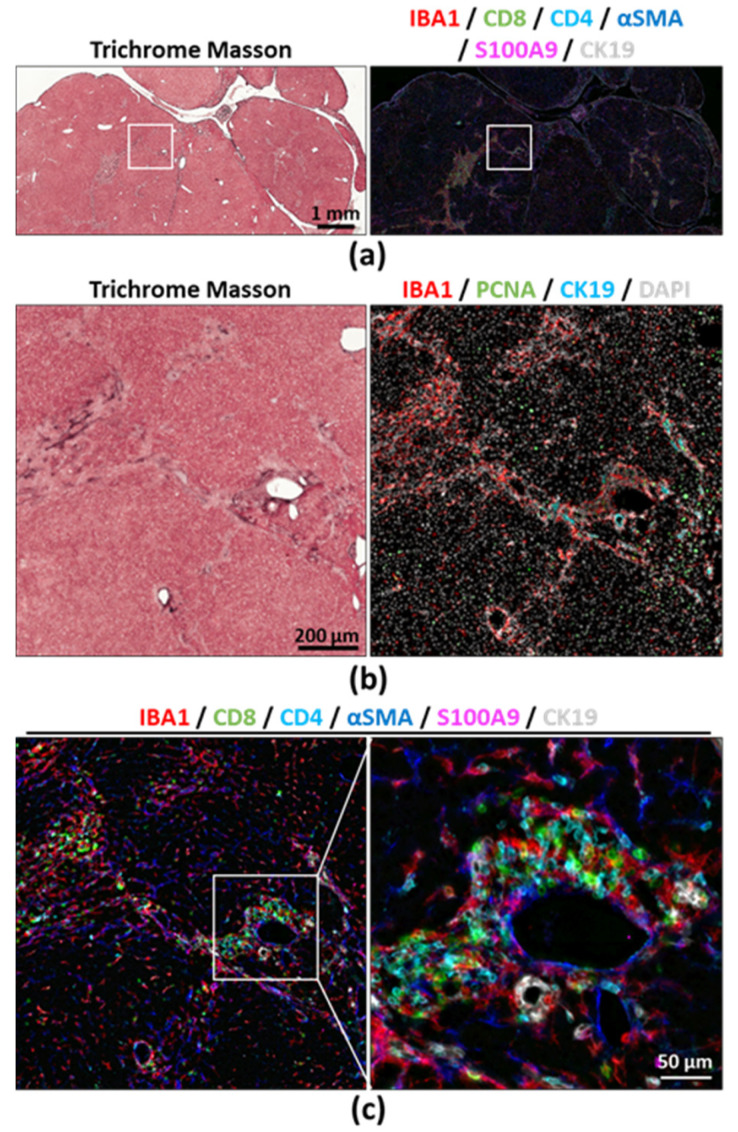
Mouse was subjected to a model of hepatocarcinogenesis sustained by chronic liver injury, consisting of a single diethylnitrosamine (DEN, 25 mg/kg intraperitoneally) injection at the age of 14 days, then at the age of 8 weeks the mouse received repeated carbon tetrachloride, twice per week for 16 weeks (CCl_4_, 0.5 mL/kg intraperitoneally) before euthanasia. (**a**) Large field scan showing Masson’s trichrome (left) and multiplex immunofluorescence acquisition (right) from a mouse FFPE liver section. The white square shows the area that was enlarged in Figure 2b,c. (**b**) enlarged area depicting Masson’s trichrome and immunofluorescence over the multiple staining cycles; (**c**) additional enlargement showing the resolution at which images were acquired. Single-channel pictures provided in Figure S5a–c.

**Figure 3:**
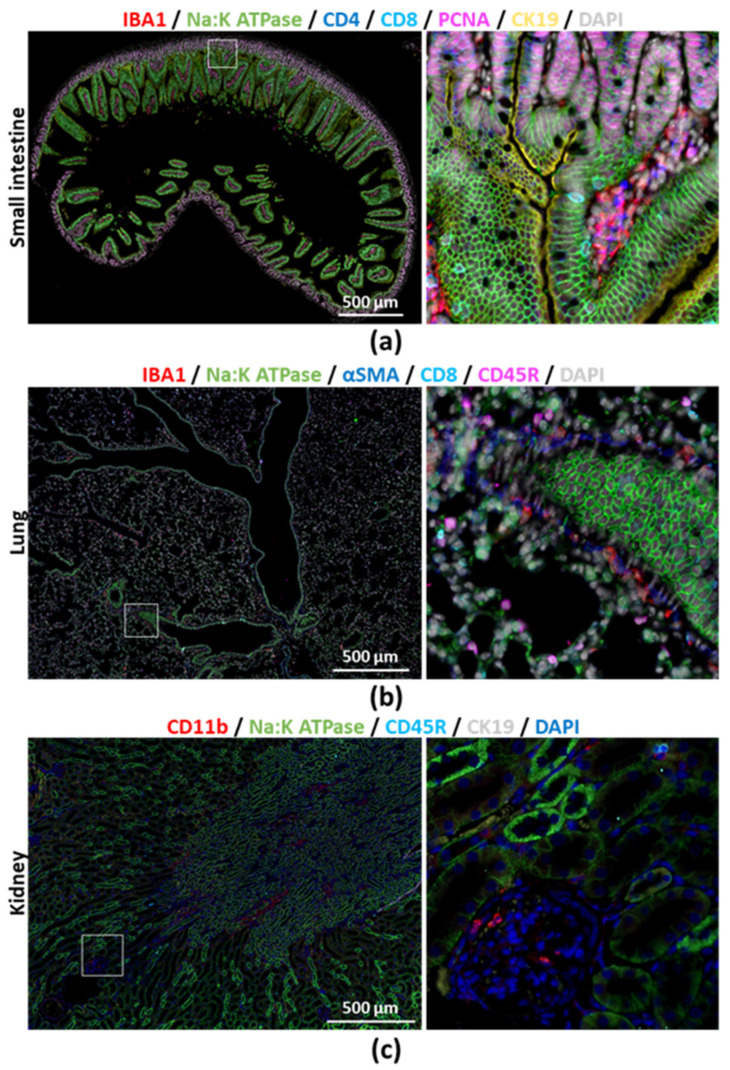
Multiple cycles of immunostaining were performed on FFPE tissue sections from (**a**) small intestine, (**b**) lung and (**c**) kidney from a healthy 8-week-old C57BL/6 J mouse. Areas delimitated with white squares in the left panels are enlarged in the right panels. Single-channel pictures provided in Figures S6–S8.

We next acquired images from large scanned areas from a mouse liver section that underwent eight immunostaining and seven stripping cycles, and from which we achieved a total of thirteen different immunostainings in addition to the nucleus staining (DAPI), followed by Masson’s trichrome (Figure 2 and Figure S5a). Even after eight cycles, DAPI images were still successfully merged and did not reveal physical tissue damage due to handling (Figure S5b). Furthermore, intact tissue and cell morphology was confirmed by Na:K ATPase immunostaining performed during the sixth cycle and showing well preserved cell membranes (Figure S5c). Large scans allowed us to visualize tissue-wide histological changes, together with a better identification of cell clusters and potential regionalization of immune cell recruitment in healthy and diseased liver upon higher magnification (Figure 2b). Remarkably, this protocol is applicable to other tissues, as we successfully performed multiplex immunostaining and large field scans on murine lung, kidney and intestines without noticing tissue damage (Figure 3a–c and Figures S6–S8).

### 2.3. Assessing Proliferation of Different Cell Compartments by Unbiased Image Processing and Quantitative Analyses

**Figure 4:**
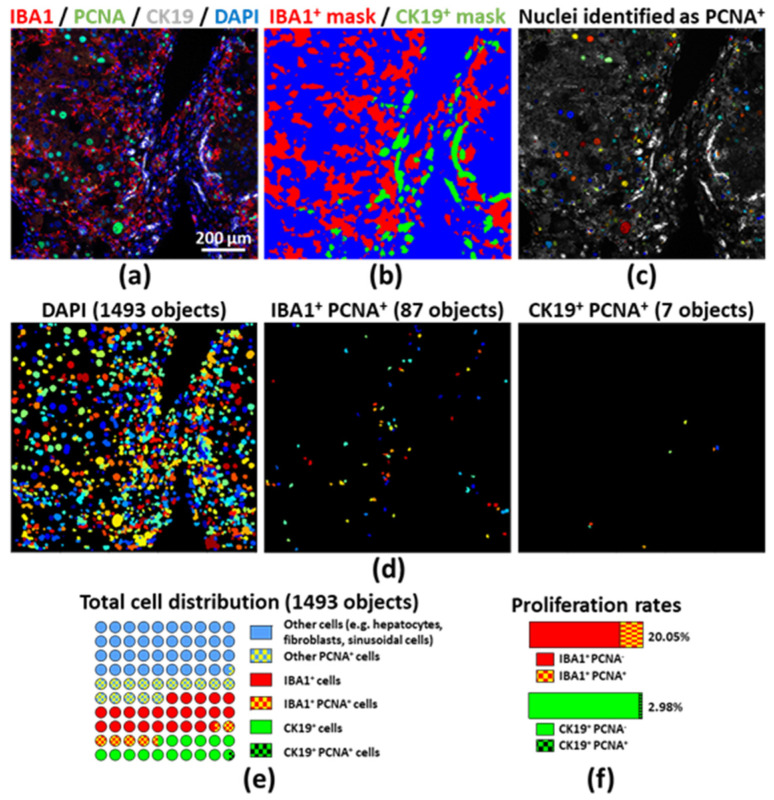
(**a**) Multiplex immunostaining was performed on a FFPE section obtained from a DEN and CCl_4_ injected mouse (fibrosis–cancer model, compare to Figure 2); (**b**) Segmentation was performed using Ilastik; (**c**) Proliferating cell nuclear antigen (PCNA)-positive nuclei were identified and labeled on the original picture by using CellProfiler; (**d**) Single nuclei from every cell, monocyte/macrophages (IBA1^+^) or ductular cells (CK19^+^) were classified and counted using CellProfiler; (**e**) Segmented cells were numbered and the chart depicts the proportions of proliferating cells with the IBA1^+^, CK19^+^ or remaining cell compartments; (**f**) ratio of PCNA^−^ versus PCNA^+^ monocytes/macrophages (upper panel) and ductular cells (lower panel). Additional masks used for this analysis shown in Figure S9.

Multiplex immunostaining may unravel histological changes; for instance, help characterize the consequences of a specific treatment or condition on the tissue microenvironment. However, this demands the “unbiased” quantitative assessment of such changes from a large and representative tissue area. Here, we illustrate data generated from a single slide, using a cyclic immunostaining protocol coupled with freely available software for image processing and analysis. One hallmark of chronic liver diseases is inflammation, notably characterized by macrophage accumulation and biliary epithelial cell-like and liver progenitor cell (or ductular cell) activation [12,13]. Figure 4 shows a liver section from a mouse that was subjected to a hepatic cancer model sustained by chronic liver damage and inflammation, induced by diethylnitrosamine (DEN) and chronic carbon tetrachloride (CCl_4_) injections [14]. We evaluated the number of macrophages and ductular cells, in conjunction with their proliferation rate, by using freeware software tools. Liver resident and monocyte-derived macrophages were stained by using an anti-IBA1 antibody, ductular cells were stained for CK19, and proliferation was evaluated with an anti-PCNA antibody (Figure 4a and Figure S9) [15]. We identified macrophages, ductular cells, as well as total and PCNA^+^ nuclei from the whole field using Ilastik, a trainable segmentation software (Figure 4b). For total, PCNA^+/−^, macrophage and ductular cell identification and counting, we further used CellProfiler (Figure 4c,d). With this data, we were able to evaluate the percentage of proliferating cells in the respective populations (Figure 4e,f). Once set, the segmentation training and the analysis pipeline may be applied to a collection of images for consistent, fast and unbiased image analysis, using established tools [16]. Depending on the combination of staining that were performed, this approach may generate very diverse data, from cell numbers to individual phenotyping or cell–cell distance (e.g., to suggest cellular interactions).

### 2.4. Large Scale Tissue Analysis: Characterizing Macrophage Compartment Alterations

**Figure 5:**
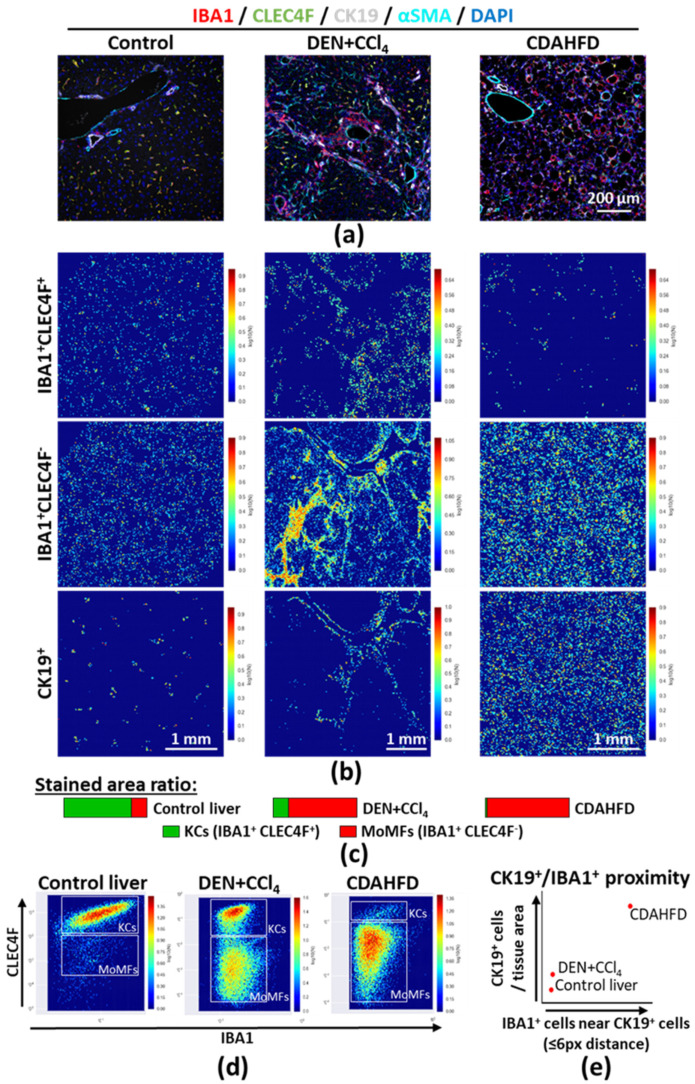
(**a**) Multiplex immunostaining was performed on mouse FFPE liver sections to identify monocyte-derived macrophages (MoMFs, IBA1^+^CLEC4F^−^) and liver resident macrophages (i.e., Kupffer cells, KCs, IBA1^+^CLEC4F^+^) in healthy, DEN and CCl_4_ injected, choline-deficient, L-amino-acid-defined, high-fat diet (CDAHFD, 12 weeks) fed mice. Single-channel pictures are provided in Figure S10a–c; (**b**) MoMFs (IBA1^+^CLEC4F^−^), KCs (IBA1^+^CLEC4F^+^), and ductular cells (CK19^+^) were segmented using Ilastik, and spatial distributions were evaluated by using CellProfiler and CellProfiler Analyst; (**c**) Area covered by IBA1^+^CLEC4F^−^ and IBA1^+^CLEC4F^+^ cells were measured in FIJI, using the masks generated in Ilastik; (**d**) Staining intensity for IBA1 and CLEC4F in individual stained cells was determined using CellProfiler; (**e**) CK19^+^ and IBA1^+^DAPI^+^ cells within 6 pixels from CK19^+^ cells were counted using CellProfiler, as described in Figure S12. Data represented as relative number of cells per total tissue area (arbitrary unit). Additional masks and enlargements provided in Figure S11a–f. Abbreviations: CDAHFD—choline-deficient; L-amino-acid-defined; high-fat diet; KCs—Kupffer cells; MoMFs—monocyte-derived macrophages.

Histological approaches, compared to cell suspension or tissue homogenate analysis, provide crucial information on tissue changes, immune-cell composition and in situ cell–cell proximity. As a use case, hepatic phagocytes mainly consist of monocyte-derived macrophages (MoMFs) and liver resident macrophages, also known as Kupffer cells (KCs). The balance between these two populations and their respective activation states tremendously regulate the immunological status of the liver, as well as tissue damage or repair [12]. Accordingly, we stained for all monocytes/macrophages (IBA1, staining monocytes, MoMFs and KCs), KCs (CLEC4F), biliary and ductular cells (CK19) and vascular cells/fibroblasts (αSMA) (Figure 5a and Figure S10). In the healthy mouse liver, KCs are located throughout the parenchyma, while MoMFs are mainly observed near the portal triad, consisting of branches of the portal vein, the hepatic artery (surrounded by αSMA^+^ cells) and the bile duct (formed by CK19^+^ biliary cells). In the DEN+CCl_4_ model, chronic injury-driven hepatocarcinogenesis is characterized by massive MoMF infiltration, fibrogenesis and ductular cell proliferation, as shown by increased IBA1, αSMA and CK19 staining, respectively. In the choline-deficient L-amino-acid-defined high-fat diet (CDAHFD) steatohepatitis model [9], steatosis is apparent by large lipid vesicles in liver parenchymal cells (hepatocytes), in some cases surrounded by MoMFs and/or fibroblasts, while KCs are largely depleted. Furthermore, ductular cells accumulate in this model (Figure 5a). While both injury models revealed increased numbers of immune and ductular cells, the location and morphology of the stained cells reflect that dramatically different processes are undergoing. Large field scanning demonstrated remarkable variations in MoMF/KC numbers and distribution (Figure S11). After segmentation and individual cell location analysis, we generated density maps and further confirmed these observations (Figure 5b and Figure S11). Moreover, IBA1 and CLEC4F stained area ratios validated this conclusion (Figure 5c). Lastly, we measured the staining intensity in individual cells and generated dot plots in CellProfiler and CellProfiler Analyst, further confirming the observation that MoMF and KC balance is altered in these models of chronic liver injury (Figure 5d). We next combined the information gathered from macrophages and ductular cells and quantified the accumulation of IBA1^+^ cells in the surrounding (≤ 6 pixels) of ductular cells. Using this method, we could also quantify the close proximity between MoMFs and ductular cells in the CDAHFD model compared to control and DEN+CCl_4_ injected mouse livers (Figure 5e). Altogether, unbiased image processing and analysis provide important factual data that may strengthen research studies based on histological characterization.

### 2.5. Defining Tissue Regions for Tumoral and Extratumoral Immune Cell Characterization

**Figure 6:**
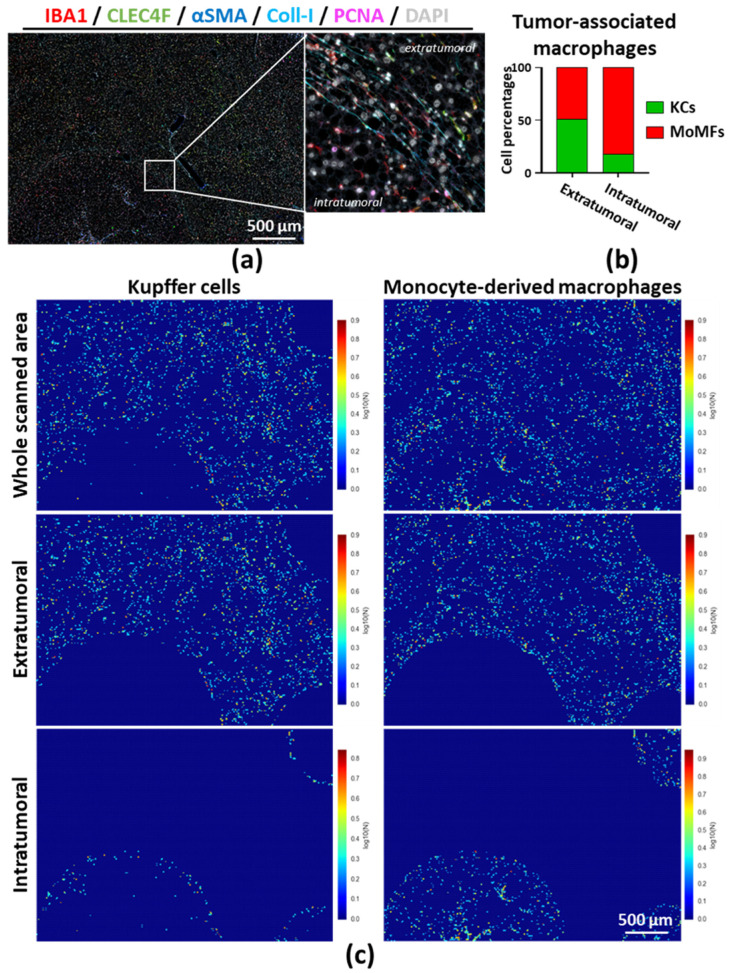
(**a**) Multiplex immunostaining was performed on a liver FFPE section from a mouse that was injected with DEN and fed with a Western diet for 16 weeks (steatohepatitis–cancer model); (**b**) Tumor and extratumoral regions defined based on PCNA and collagen-1 stained areas and Kupffer cells (KCs, IBA1^+^CLEC4F^+^); monocyte-derived macrophages (MoMFs, IBA1^+^CLEC4F^−^) numbered as described above. Graph represents the relative contribution of KCs and MoMFs in the macrophage compartment of each region; (**c**) MoMFs and KCs segmented using Ilastik; and spatial distributions were evaluated by using CellProfiler and CellProfiler Analyst. Additional masks provided in Figure S13a,b. Abbreviations: KCs—Kupffer cells; MoMFs—monocyte-derived macrophages.

During progression of chronic liver diseases from premalignant fibrosis/cirrhosis to hepatocellular carcinoma, the histology is profoundly altered, and regions of interest can be defined, e.g., fibrotic septa, necrotic areas or tumor regions. We used an FFPE liver section from a mouse that was subjected to a DEN + Western diet (DEN+WD)-induced liver cancer and performed multiplex immunostaining using five antibodies. As shown in Figure 6a and Figure S13a,b, the simultaneous staining of Collagen type I and PCNA allowed to delineate the tumor areas in the liver. More specifically, we considered as tumoral areas collagen-I encapsulated structures containing PCNA-positive hepatocyte-like nuclei. IBA1 and CLEC4F staining also revealed the distribution of MoMFs and KCs within the tumors and the surrounding tissues. By using a similar combination of trainable segmentation and cell identification as described above, we were able to quantify the relative contribution of MoMFs and KCs to the macrophage compartments of the distinct regions (Figure 6b,c and Figure S13c). KCs were located in the peritumoral area, whereas MoMFs were homogeneously present throughout the tumor (Figure 6c).

## 3. Discussion

We herein described a complete workflow for the implementation of a simple, cost-effective and reproducible multiplex immunostaining protocol followed by rigorous and unbiased image analysis. This method can be applied to the widely available FFPE archival samples, whether from clinical patient cohorts or animal models. We believe this protocol offers incredible potentials in terms of better understanding histology in the large sense, as it can depict cell phenotypes, extracellular matrix changes and inflammatory processes. This approach may, for instance, be useful for tumor stage assessment and immune microenvironment or immune cell infiltration characterization. It could also be easily extended to T cell subsets and specific markers such as Programmed cell death protein 1 (PD-1) or cytotoxic T-lymphocyte-associated protein 4 (CTLA-4). Furthermore, it is relatively inexpensive and should thus be applicable in most if not all laboratories currently performing immunostaining on a regular basis. The high flexibility of this protocol allowed the rapid validation of antibodies that were already in stock in our laboratory and used in conventional immunohistochemistry, without any antibody modifications (e.g., fluorochrome or metal conjugation). Importantly, this protocol relies on an efficient antibody elution that has been previously described and was integrated into our immunohistochemistry protocols [17,18]. We here indistinctly used mono- and polyclonal antibodies. Polyclonal antibodies directed against several epitopes of the same antigen, may help amplify weak staining signal. However, monoclonal antibodies should be preferred for establishing diagnostic procedures and for reducing the risk of cross-reaction. Noteworthy, this method easily runs on high-end microscopes that allow rapid and high-resolution whole slide imaging.

A similar 2-mercaptoethanol/SDS stripping approach was used to characterize bone marrow immune cell populations [19]. In this study, the authors thoroughly demonstrated the feasibility of multiplex immunostaining on bone marrow tissue without significantly affecting antigenicity or tissue morphology. However, this report did not elaborate much on image analysis and data generation, and a commercial software was used [19]. Gerdes et al. used a patented (U.S. patent 7,741,045) alkaline oxidation-based chemical reaction for irreversible inactivation of antibody-conjugated fluorophores, by which the antibody–fluorophore complex remains on the tissue [20]. For this approach, the use of conjugated primary antibodies is appropriate, thus reducing hands-on time requirements. Contrastingly, we recommend the use of secondary antibodies with stripping methods, in order to amplify the signal on the one hand and provide a high flexibility on the other hand. Furthermore, the authors stated that they applied up to 100 cycles of dye-inactivation to show that antigen integrity was not affected by the chemicals; however, they did not stain the same antigen more than once on a single slide. This is, expectedly, challenging because the antigens should be already be saturated by bound antibodies from the previous cycle. It is conceivable that distinct antibodies may compete for binding to neighboring amino acid positions on the target protein (e.g., total versus phosphorylated proteins). By using the 2-mercaptoethanol/SDS antibody stripping, we are able to stain the same antigen several times, using virtually any dye, which provides more flexibility. This may be particularly valuable, if, for some reasons, staining(s) have to be repeated or different combinations of antibodies must be used. Importantly, Bolognesi et al. reported that the 2-mercaptoethanol/SDS-mediated antibody stripping moderately affected staining intensity over ten staining cycles (ranging within 10% above or below the initial staining). Thus, the tissue and antigen qualities do not seem to be notably altered along the protocol [18].

Multiplexing strengthens the data generated by immunohistochemistry by limiting false positive staining interpretation, e.g., by revealing nonspecific staining that is repeatedly observed over several cycles or secondary antibody cross-reaction and does not have any relevant physiological meaning. By using Ilastik, for instance, it is feasible not only to segment a cell population based on a single marker-expression, but on the co-expression of several markers, including DAPI, that would consequently eliminate irrelevant signal. This is particularly relevant when performing immunohistochemistry on FFPE samples or when dealing with intrinsic tissue components known to be autofluorescent [21,22]. A systematic approach using multiplex immunostaining could very well be used for screening and preliminary characterization of poorly described patient conditions or experimental models. It can also be used to validate, in a large number of samples, the presence and localization of distinct cellular subpopulations or phenotypes that have been identified by “omics” technology such as single-cell RNA sequencing [23,24]. Another crucial advantage of the herein proposed method is that there is no particular pre-analytic tissue handling required, making it possible to apply our method for retrospective analysis of any existing archival FFPE samples.

Alternative methods have been described or are commercially available, allowing for the visualization of multiple cell types within the same sample [6]. Imaging mass cytometry for instance, would theoretically allow for about 100 markers to be visualized at once at the single cell level, which may provide unprecedented information on cell phenotype [25]. However, one major limitation of this technique is that currently, image acquisition is time consuming and only allows for the visualization of a relatively small tissue area and a limited number of samples. Antibody-based approaches, such as our protocol, may also be combined with in situ hybridization for the simultaneous proteome and transcriptome analysis [26,27,28].

Flow cytometry (FC) is the method of reference for circulating blood cell characterization. Notably, multiplex immunohistochemistry differs yet complements FC-based approaches for tissue infiltrating immune cell characterization, on several aspects. On one hand, FC implies the need for tissue dissociation and the loss of spatial information or contextualization. Depending on the tissue dissociation method, some cell types may be over-represented in the final cell suspension, while others may have been depleted. On the other hand, FC may provide valuable information on physiological processes in live cells. Current FC methods, such as multi-laser spectral FACS or mass cytometry (i.e., CyTOF/cytometry by time of flight) allow the simultaneous assessment of many (>30) antibodies at a single-cell resolution, which can provide a very comprehensive protein expression profile. In comparison, tissue sectioning, as the basis for subsequent immunohistochemistry, will inherently show a limited area of the tissue of interest and may thereby introduce a bias. However, tissue section analysis provides key information on immune cell distribution across the tissue and possible immune—parenchymal cell-to-cell interactions that would need to be further demonstrated in vitro, for instance.

Fluorescence microscopy offers great advantages over bright field image acquisition, by generating images that can readily be analyzed by the appropriate software. While it may introduce artefacts (e.g., auto fluorescence of lipid droplets in the steatohepatitis model), adequate image processing may greatly enhance the image quality and thus, help generate more robust data. Image preprocessing include tile stitching and background subtraction and autofluorescence subtraction (Figures S13 and S14, respectively) [29]. Furthermore, image analysis and data generation can be performed using open source software, and a significant amount of data may be generated from multiplex immunostaining using bioinformatics resources [30,31,32].

As a conclusion, distinct methods for multiplexing have their own advantages and pitfalls that one must consider during project design. In our manuscript, we not only report on multiplex immunostaining, but also on antibody validation data, image processing and analysis with direct implications in the field of liver pathophysiology that could be adapted to numerous other research areas. Of importance, the data herein presented have been acquired using conventional microscopes and freeware, thus granting access to the method to virtually any laboratory routinely performing immunostaining. In the era of the “omics” studies, classical immunohistochemistry approaches that allow for the visualization of one to six markers may have significant limitations. Multiplex immunohistochemistry combined with machine–learning and automated image analysis will improve our understanding of tissue (patho)physiology in an unprecedented manner. Automated image analysis pipelines together with robust and quantitative data generation will also allow for fast and reproducible analysis that is not prone to experimenter or pathologist variability.

## 4. Materials and Methods

### 4.1. Tissue Preparation

Archival human and mouse samples were used to illustrate the method herein described. The human study was conducted in accordance with the Declaration of Helsinki, and the protocol was approved by the Ethics Committee of Charité-Universitätsmedizin Berlin (Project EA2/091/19). All animal experiments were conducted according to German Laws for Animal Protection and the National Institute of Health Guidelines for Care and Use of Laboratory Animals and approved by the State Agency for Nature, Environment and Consumer Protection (LANUV) of North Rhine-Westphalia, Germany (Nos. 84-02.04.2015.A554; 84-02.04.2013.A054; 84-02.04.2017.A061). As a mouse model of primary liver cancer in fibrosis, mice received a single intraperitoneal diethylnitrosamine (DEN) injection (25 mg/kg body weight) at the age of 2 weeks, then repeated carbon tetrachloride (CCl_4_, 0.5 mL/kg body weight) injections from the age of 8 weeks, twice per week for 16 weeks [14]. Alternatively, DEN injection was followed by a 16-week Western-diet feeding (Ssniff EF+D88137 med. +1,25% cholesterol, Soest, Germany) from the age of 8 weeks. As a liver fibrosis model, CCl_4_ was injected twice per week into 8-week-old mice for 8 weeks. To study mechanisms of steatohepatitis, choline-deficient, L-amino-acid-defined, high-fat diet (CDAHFD) was given for 12 weeks [9]. Tissues were fixed, dehydrated and paraffin-embedded in routine conditions. Four-micrometer-thick tissue sections were prepared for immunostaining. Slides were de-paraffinized in two successive baths of xylene and rehydrated in gradually decreasing ethanol baths (95%, 80%, 70% and 50%) for 5 min each, followed by immersion in distilled water.

### 4.2. Antigen Retrieval

Antigen retrieval was performed by immersing the slides in preheated citrate (pH 6.0, Thermo Fisher, Waltham, MA, USA) or EDTA (pH 9.0, Novus Biologicals, Centennial, CO, USA) buffer for 20 min, in a water bath set at +100 °C followed by a cool down phase for 30 min on the bench.

### 4.3. Blocking

Nonspecific antibody binding was prevented by incubating mouse tissues in 2% normal goat serum (Thermo Fisher) and human tissues in 2.5% horse serum (Vector BioLabs, Malvern, PA, USA), for one hour at room temperature. Auto-fluorescence was reduced by incubating the tissue sections in Image-iT FX Signal Enhancer (Thermo Fisher) for 30 min at room temperature.

### 4.4. Primary and Secondary Antibody Incubation

The list of antibodies used in this study is provided in Table S1. Antibodies were diluted in PBS supplemented with 1% *w*/*v* bovine serum albumin (Sigma-Aldrich, Saint-Louis, MO, USA). Primary antibodies were incubated overnight at +4 °C (ca. 16 h), secondary antibody mix were prepared depending on the primary antibody(ies) host species, then applied on the tissue sections for one hour at room temperature. When necessary, VectaFluor Excel Amplified DyLight 488 anti-rabbit IgG Kit (Vector BioLabs) was used for signal amplification prior to additional secondary antibody incubation, following manufacturer’s instructions. To verify that the secondary antibodies do not cross-react, negative controls in which primary antibodies are omitted should be included.

### 4.5. Nucleus Staining

Following secondary antibody incubations, slides were incubated in PBS containing 2-µg/mL DAPI (Sigma-Aldrich) for 5 min at room temperature (Note S1).

### 4.6. Antibody Stripping

Antibody elution was performed with the 2-mercaptoethanol/SDS (2-ME/SDS) method as previously described with slight changes [17,18]. Briefly, slides were immerged in distilled water just after imaging until the cover glass slid down (ca. 30 min), then incubated in stripping buffer for 30 min at +56 °C. The stripping buffer consisted of 62.5-mM Tris-HCl pH 6.8 (Bio-Rad, Hercules, USA), 2% *w*/*v* SDS (Rockland Immunochemicals, Limerick, PA, USA) and 114.4-mM β-mercaptoethanol (Sigma-Aldrich) prepared in distilled water. One milliliter of stripping buffer was prepared as follow under a chemical hood: 675 µL distilled water, 125 µL 0.5-M Tris-HCl (pH 6.8), 200 µL 10% *w*/*v* SDS, 8 µL β-mercaptoethanol. Afterwards, slides were washed for 3x 20 min in PBS containing 1% Tween-20, under agitation (Notes S2 and S3).

### 4.7. Counterstaining

Masson’s trichrome was performed at the end of the sequential immunostaining and following fluorescence signal acquisition, according to the manufacturer’s instructions (Abcam, Cambridge, UK) (Note S4).

### 4.8. Imaging

Slides were extensively washed in PBS then deionized water and mounted with VectaMount AQ Aqueous Mounting Medium (Vector BioLabs) just prior to imaging. Single field images were acquired on a Zeiss Observer.Z1 microscope (Carl Zeiss, Oberkochen, Germany). Whole slide scanning was performed on a Zeiss Axio Observer 7. Image acquisition was performed in the same regions over the successive days of the protocol. Precise image alignment is described below.

### 4.9. Image Preprocessing

Following acquisition, large field scanned images were stitched, and a background subtraction was performed with the default settings using the ZEISS software ZEN 3.1 (blue edition) (Figure S14). Single channel grayscale pictures were further processed in FIJI [33]. For CDAHFD pictures, autofluorescence due to diet compounds was subtracted from the single-channel pictures using the image calculator module, when necessary (Figure S15a,b). Collagen staining was extracted from the Masson’s trichrome acquired image with the “color threshold” selection tool. Color merges were generated as appropriate for illustration purposes (Note S5).

### 4.10. Image Alignment

Single-channel pictures acquired the same day were put together in a hyperstack, making sure the DAPI channel was in channel 1. Hyperstacks were concatenated, and alignment was performed using the FIJI HyperStackReg V5.6 plugin with the affine setting, using DAPI (channel 1) as a reference for alignment. Bright-field images (e.g., Masson’s trichrome) were aligned using the Align Image by line ROI plugin. Following alignment, images were cropped to remove non-merging areas, and single-channel pictures were saved separately (Note S6).

### 4.11. Image Analysis

Binary masks were generated using the trainable classification software Ilastik (v 1.3.3) [34]. Stained area was measured using FIJI. Cell identification, counting, distribution, intensity measurement, and co-staining expression analysis were performed on CellProfiler v3.1.9 and CellProfiler Analyst (Notes S7 and S8) [35,36]. The CellProfiler project we used in Figure 5 are provided in Appendix A (Note S9). Neighbor analysis was performed on CellProfiler by expanding CK19^+^ objects by 10 pixels, then counting IBA1^+^ cells within this distance (Appendix B, Note S9) [16].

## 5. Conclusions

Characterizing the tissue immune-cell composition in premalignant conditions as well as in the tumor environment can provide important information that may influence the individual patient management or guide therapeutic decisions. Traditional immunohistochemistry approaches that allow for the visualization of one to six markers may not be able to capture the level of granularity that is desired in the era of modern immune oncology. We herein present an optimized protocol for multiplex immunohistochemistry, which—combined with machine–learning and automated image analysis—can improve our understanding of tissue (patho)physiology.

## Figures and Tables

**Table 1:** Typical multiplex immunostaining workflow.

Timeline	Steps Performed
Day 1	Deparaffinization
	Antigen retrieval
	Rinse 3 × 5 min in PBS
	Nonspecific fluorescence elimination
	Rinse 3 × 5 min in PBS
	Nonspecific binding blocking
	Rinse 3 × 5 min in PBS
	Primary antibody incubation
Days 2 to x	Rinse once in PBS-T 0.1%
	Rinse 2 × 5 min in PBS
	Secondary antibody incubation
	Rinse once in PBS-T 0.1%
	Rinse 2 × 5 min in PBS
	Nucleus staining
	Rinse 3 × 5 min in DI water
	Aqueous mounting
	Imaging
	Slides immerged in DI water and cover glass removal
	Antibody stripping
	Rinse 3 × 5 min in DI water
	Antigen retrieval
	Rinse 3 × 5 min in PBS
	Primary antibody incubation
Final day	Rinse once in PBS-T 0.1%
	Rinse 2 × 5 min in PBS
	Secondary antibody incubation
	Rinse once in PBS-T 0.1%
	Rinse 2 × 5 min in PBS
	Nucleus staining
	Rinse 3 × 5 min in DI water
	Aqueous mounting
	Imaging
	Slides immerged in DI water and cover glass removal
	Masson’s trichrome
	Permanent mounting
	Imaging

Abbreviations: DAPI—4′,6-diamidino-2-phenylindole; DI water—deionized water; PBS—phosphate-buffered saline; PBS-T—phosphate-buffered saline 0.1% Tween20.

## Supplementary Materials

The following are available online at https://www.mdpi.com/2072-6694/12/9/2449/s1, Notes S1–S9, Figure S1: General workflow for multiplex immunostaining and data generation, Figure S2: Multiple cycles of immunostaining do not damage the tissue and DAPI may be used for image alignment, Figure S3: Single-channel pictures from Figure 1a, Figure S4: Single-channel pictures from Figure 1b, Figure S5: Multiplex immunostaining combined with large area scanning, Figure S6: Single-channel pictures from Figure 3a, Figure S7: Single-channel pictures from Figure 3b, Figure S8: Single-channel pictures from Figure 3c, Figure S9: Image processing steps used in Figure 4, Figure S10: Single-channel pictures from Figure 4g, Figure S11: Masks used for cell density map, Figure S12: CK19^+^ cell neighbor analysis, Figure S13: Single-channel pictures and masks used for tumor region analysis, Figure S14: Stitching and background subtraction following large field scanning greatly enhance image quality, Figure S15: Autofluorescence subtraction may enhance image quality, Table S1: Antibodies used in this study.

## Appendix A

CellProfiler pipeline used in Figure 5.

## Appendix B

CellProfiler pipeline used in Figure 6.
