# Supplementary material for: Deciphering the Immune Microenvironment on A Single Archival Formalin-Fixed Paraffin-Embedded Tissue Section by An Immediately Implementable Multiplex Fluorescence Immunostaining Protocol
Source: Cancers (Basel). 2020 Aug 28;12(9):2449. doi: 10.3390/cancers12092449 (PMC7565194; doi:10.3390/cancers12092449)
Supplement: Supplementary file 1 [file cancers-12-02449-s001.zip › cancers-883269-supply.pdf]

Supplementary Materials

Deciphering the Immune Microenvironment on A Single Archival Formalin-Fixed Paraffin-Embedded Tissue Section by An Immediately Implementable Multiplex Fluorescence Immunostaining Protocol

Adrien Guillot, Marlene Sophia Kohlhepp, Alix Bruneau, Felix Heymann and Frank Tacke

Supplementary Notes

Note S1: An intense and clear DAPI staining was key to image alignment after sequential image acquisition in our set-up. Alternatives may be used, as long as there is a constant feature that allows for image alignment across the experiment.

Note S2: In rare cases, if antibody elution was not efficient (e.g., very strong signal from the previous cycle) an additional stripping cycle may be performed.

Note S3: To prevent any issue due to residual staining and to verify antibody stripping, immunostaining expected to colocalize shall not be performed in two consecutive cycles using the same secondary antibody.

Note S4: After the last cycle of sequential fluorescent immunostaining, brightfield tissue staining may be performed. We have regularly stained the slides with Hematoxylin-Eosin and Trichrome Masson.

Note S5: Autofluorescence or any "targeted" image alterations must be done with extreme rigorousness and raw pictures should be shown beside.

Note S6: Image alignment and tissue integrity over repeated staining cycles may be checked by merging consecutive DAPI pictures (Figures S2, and S5b).

Note S7: CellProfiler benefits from a very active and supportive community, and a considerable amount of information may be found online (https://cellprofiler.org/).

Note S8: We chose to use FIJI, Ilastik and CellProfiler for their diverse plugins, extensive support communities and ease of use. A number of very valuable open source and commercial alternatives are available [1–5].

Note S9: Download the latest version of CellProfiler at this link: https://cellprofiler.org/releases, and older releases at: https://cellprofiler.org/previous-releases. We ran the provided CellProfiler projects on version 3.1.9. After installing the software, go to File > Open project, and select the appropriate cpproj file (Appendix A or Appendix B).

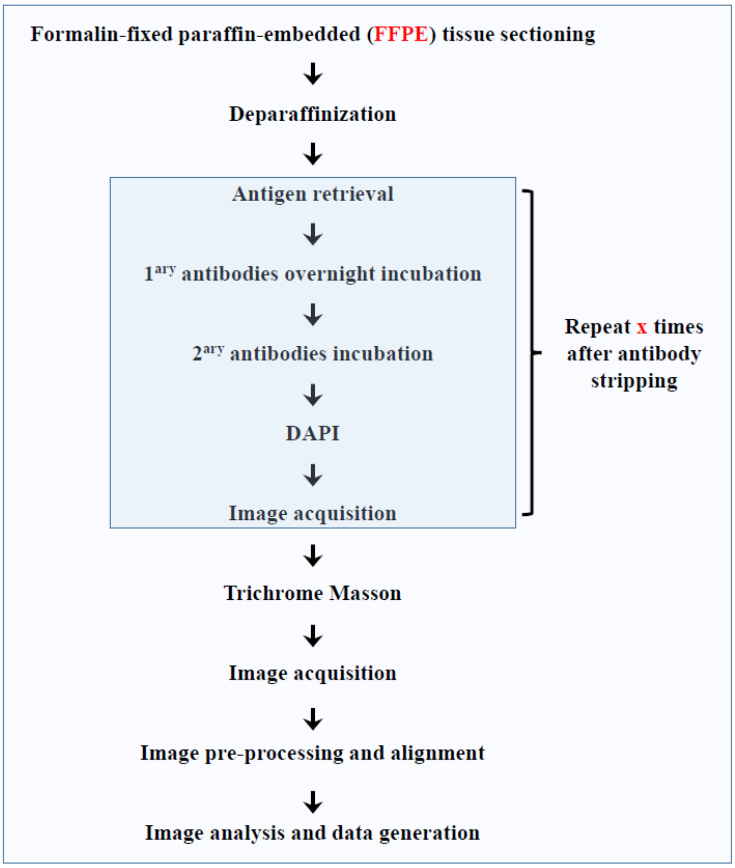

Figure S1. General workflow for multiplex immunostaining and data generation. This chart depicts the generic workflow one can apply from tissue collection to data generation.

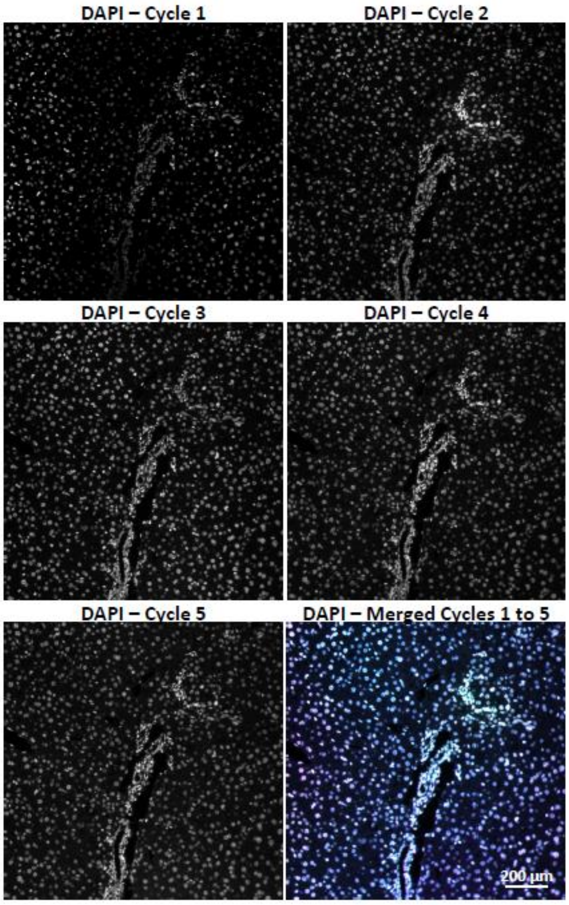

Figure S2: Multiple cycles of immunostaining do not damage the tissue, and DAPI may be used for image alignment

Figure S2. Multiple cycles of immunostaining do not damage the tissue, and DAPI may be used for image alignment. A single FFPE mouse liver section has been subjected to multiple cycles of stripping and immunostaining as depicted in Figure S1. At each cycle, DAPI image was recorded. At the end of the experiment, all DAPI pictures were aligned and merged together using FIJI (bottom right panel).

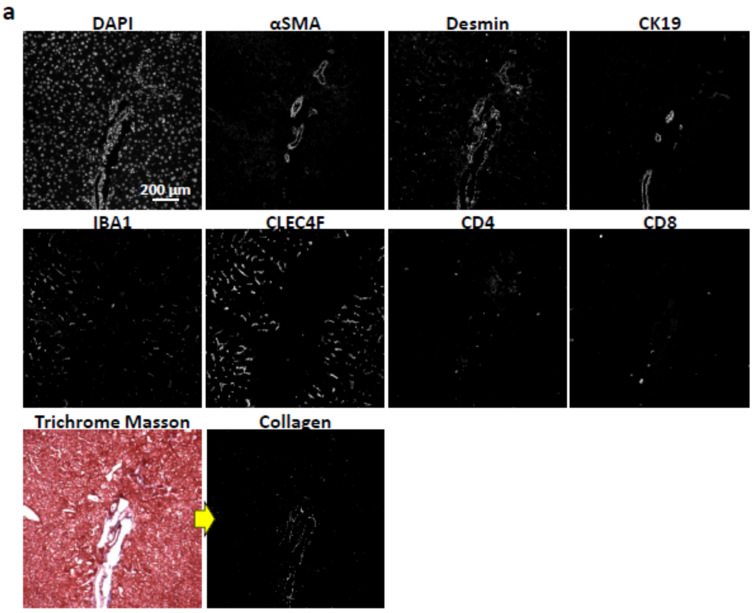

Figure S3a: Single channel pictures from Figure 1a

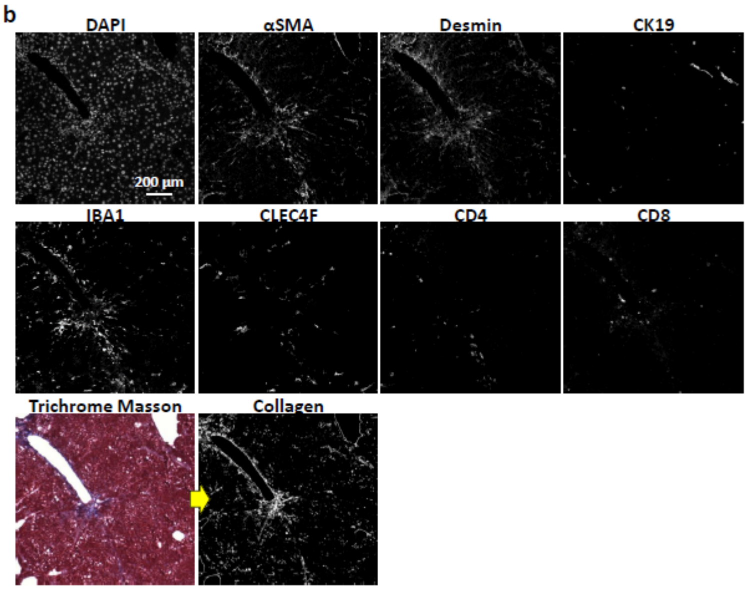

Figure S3b: Single channel pictures from Figure 1a

Figure S3. Single channel pictures from Figure 1a. (**a**) Healthy control mouse liver and (**b**) CCl_4 injected. Single immunostaining pictures are depicted in grayscale. The collagen staining (dark blue) from the Trichrome Masson was extracted using FIJI.

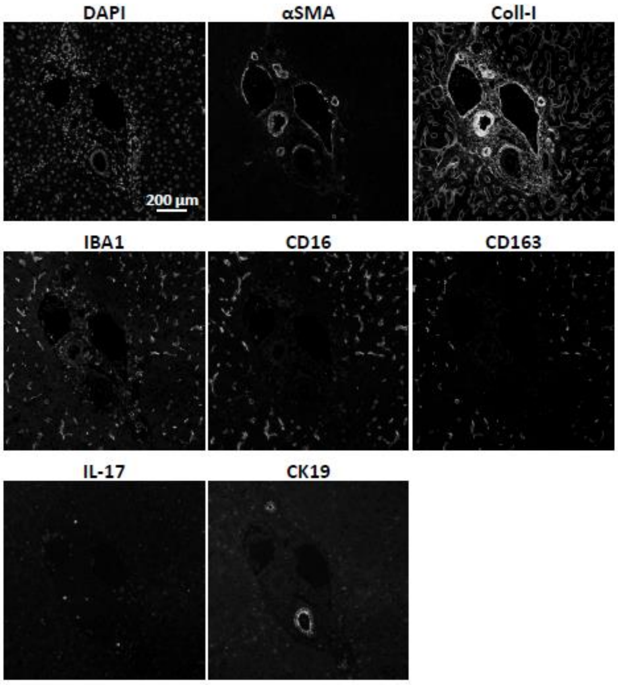

Figure S4a: Single channel pictures from Figure 1b

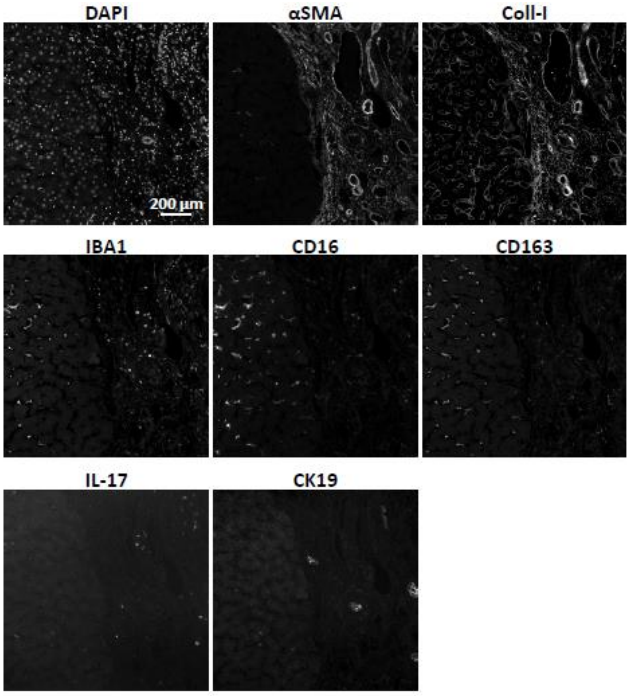

Figure S4b: Single channel pictures from Figure 1b

Figure S4. Single channel pictures from Figure 1b. (**a**) Healthy human liver and (**b**) liver resection from a patient suffering from primary sclerosing cholangitis. Single immunostaining pictures are depicted in grayscale. In this Figure, collagen staining was obtained by using a primary antibody directed against type I collagen.

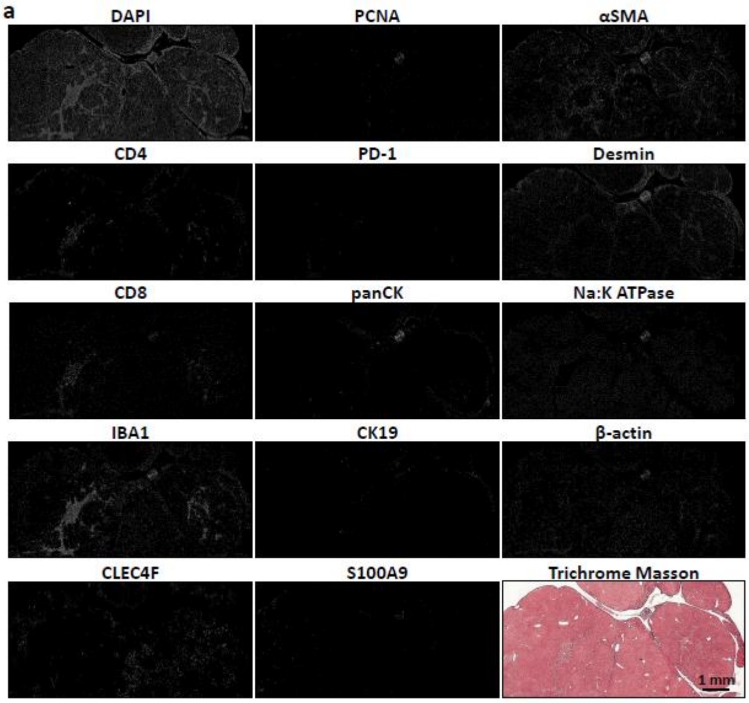

Figure S5a: Multiplex immunostaining combined with large area scanning

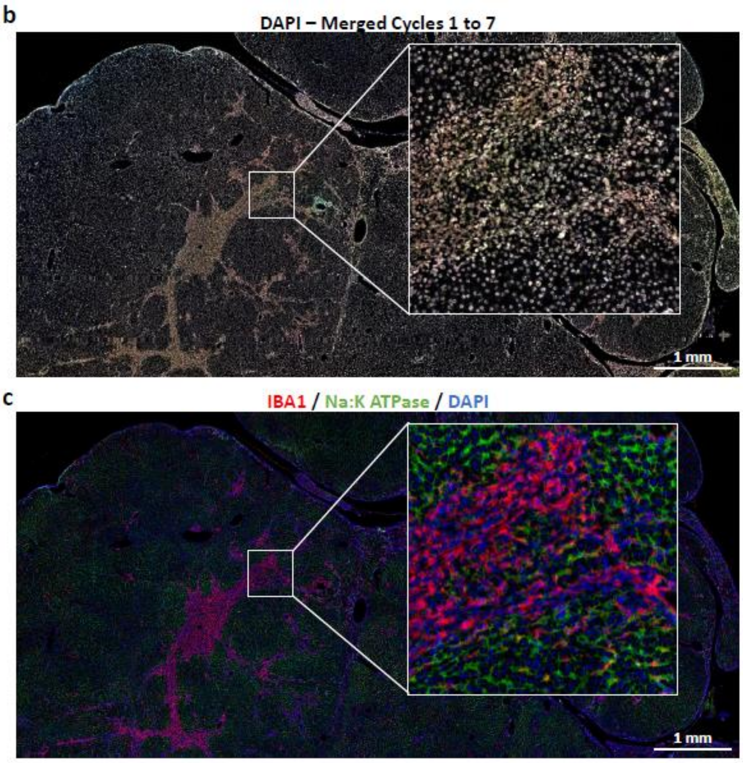

Figure S5b, c: Multiplex immunostaining combined with large area scanning

Figure S5. Multiplex immunostaining combined with large area scanning. (**a**) Single immunostaining pictures from Figure. 2a,b. (**b**) DAPI acquired images from 7 immunostaining cycles were merged together. Insert shows an enlarged area. (**c**) IBA1 (red), Na:K ATPase (green) and DAPI (blue) pictures were merged.

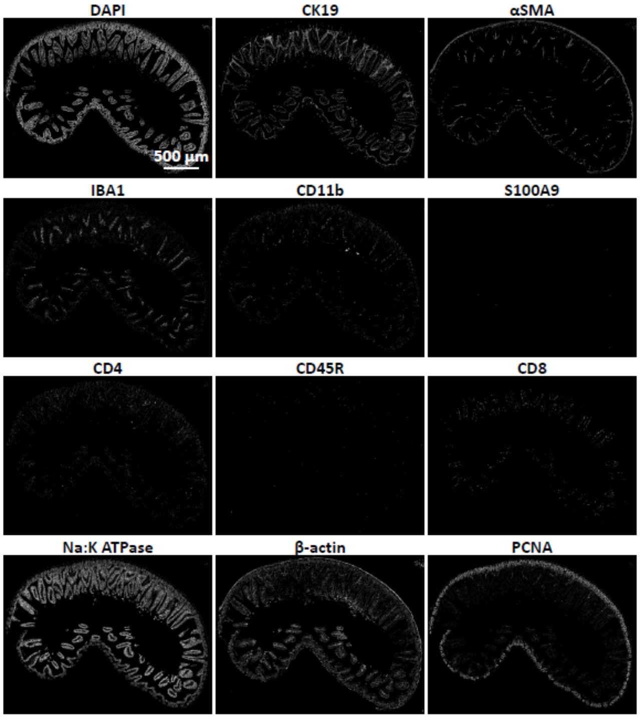

Figure S6a: Single channel pictures from Figure 3a

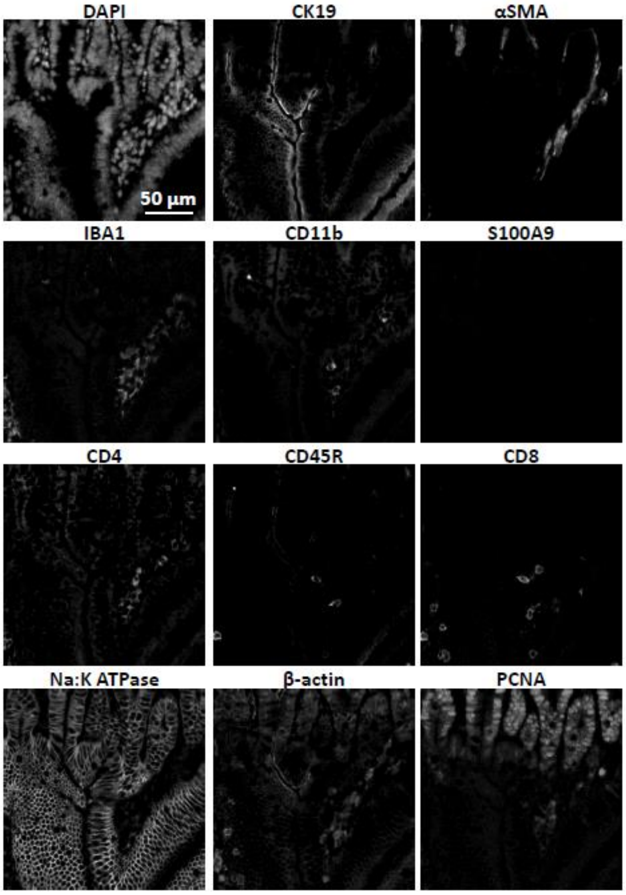

Figure S6b: Single channel pictures from Figure 3a

Figure S6. Single channel pictures from Figure. 3a. Single immunostaining pictures from (**a**) the whole scanned area or (**b**) an enlarged area, are depicted in grayscale.

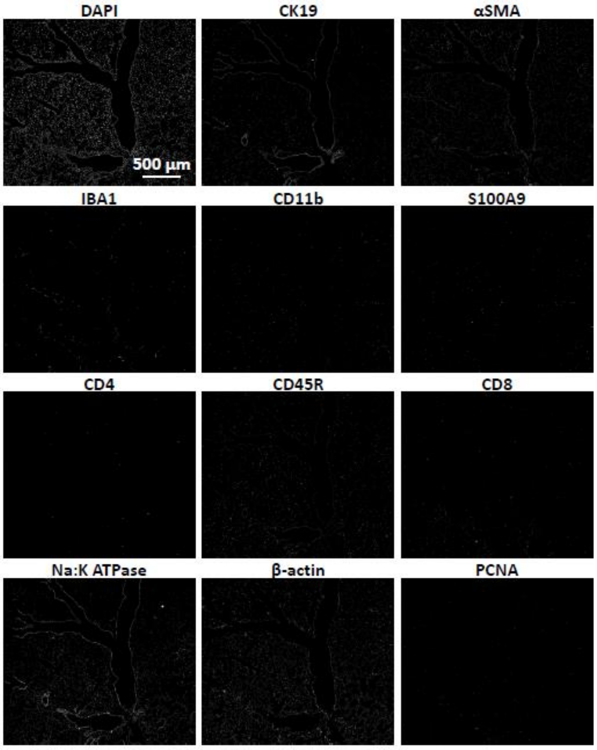

Figure S7a: Single channel pictures from Figure 3b

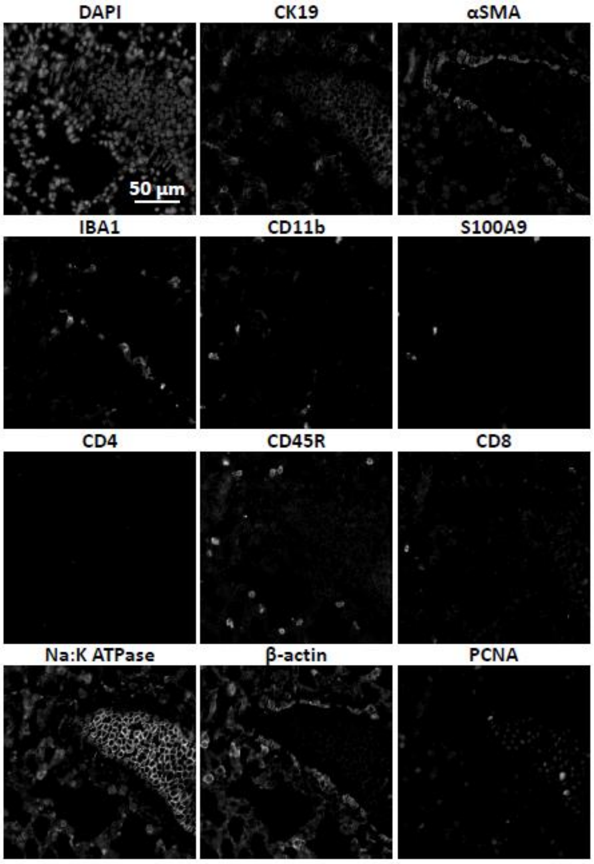

Figure S7b: Single channel pictures from Figure 3b

Figure S7. Single channel pictures from Figure 3b. Single immunostaining pictures from (**a**) the whole scanned area or (**b**) an enlarged area, are depicted in grayscale.

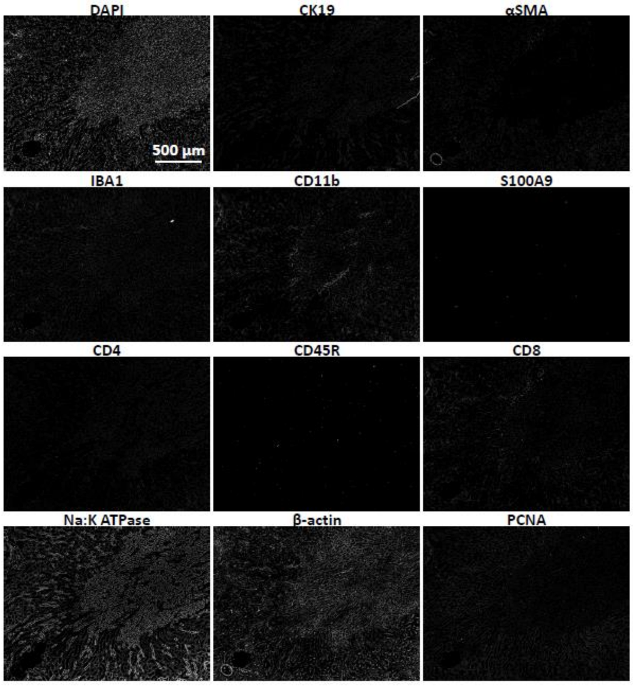

Figure S8a: Single channel pictures from Figure 3c

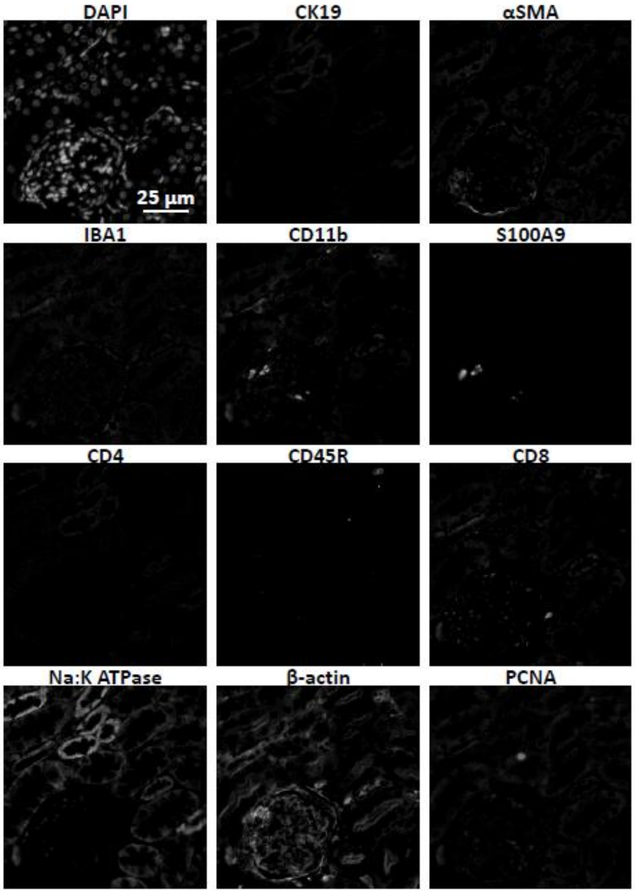

Figure S8b: Single channel pictures from Figure 3c

Figure S8. Single channel pictures from Figure. 3c. Single immunostaining pictures from (**a**) the whole scanned area or (**b**) an enlarged area, are depicted in grayscale.

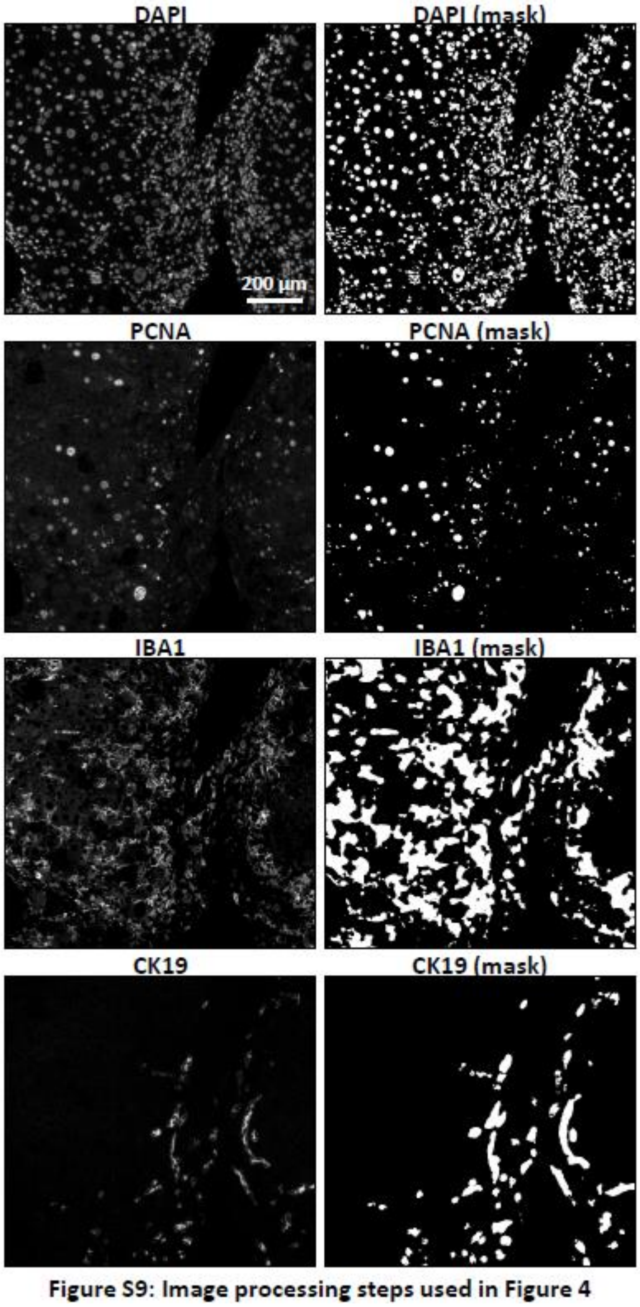

Figure S9. Image processing steps used in Figure 4. Single immunostaining pictures are depicted in grayscale (left panels). Corresponding masks generated by using Ilastik are shown in the right panels.

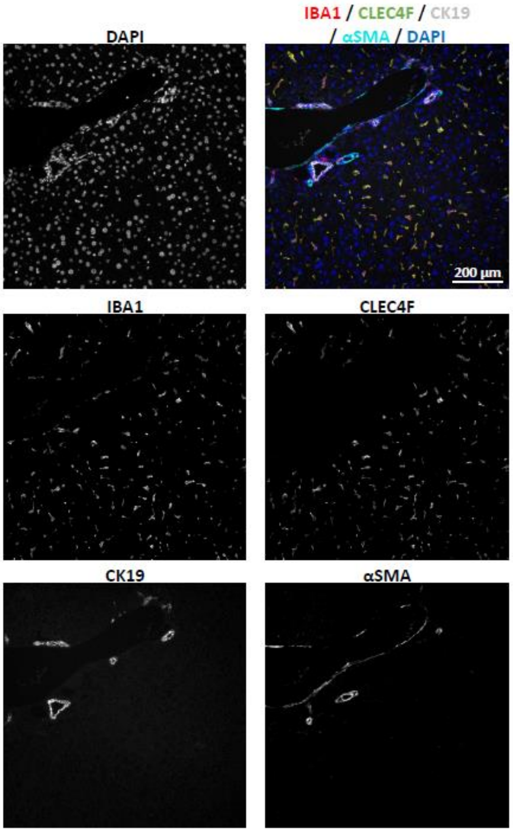

Figure S10a: Single channel pictures from Figure 5a

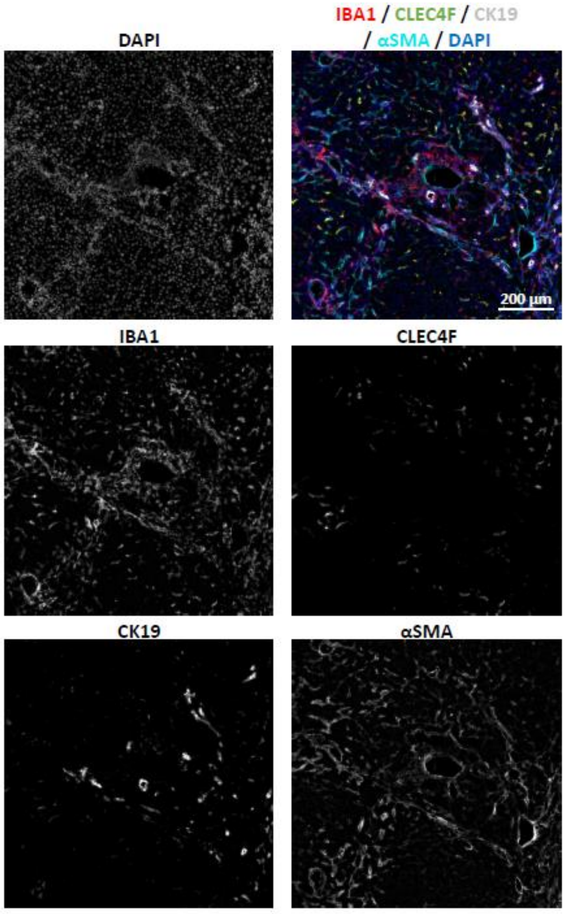

Figure S10b: Single channel pictures from Figure 5a

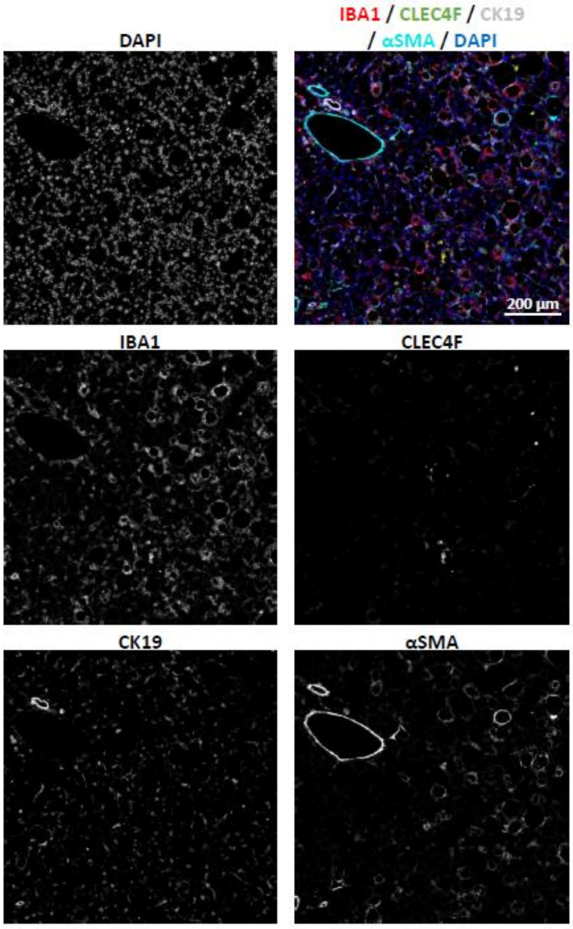

Figure S10c: Single channel pictures from Figure 5a

Figure S10. Single channel pictures from Figure 4g. Single immunostaining pictures from (**a**) healthy, (**b**) DEN and CCl4 injected, and (**c**) CDAHFD fed mice, are depicted in grayscale.

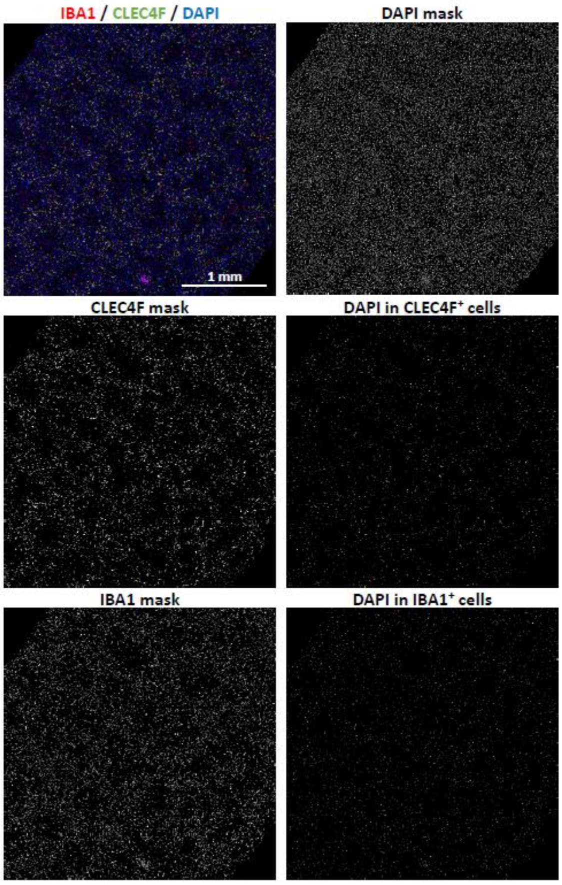

Figure S11a: Masks used for cell density map

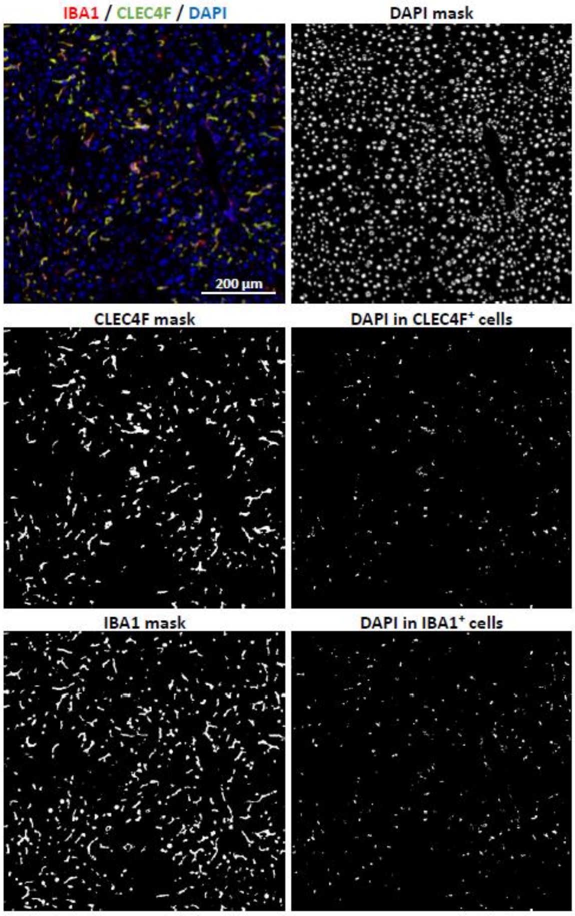

Figure S11b: Masks used for cell density map

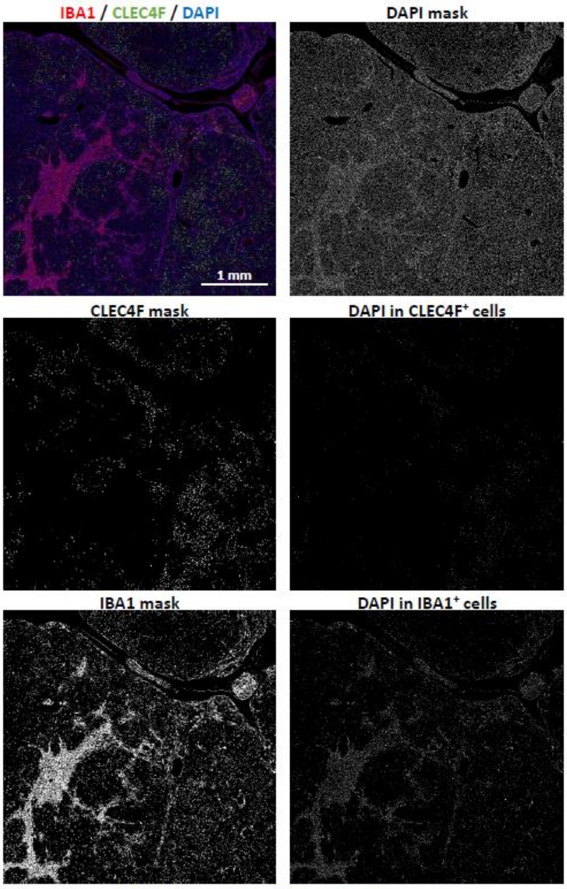

Figure S11c: Masks used for cell density map

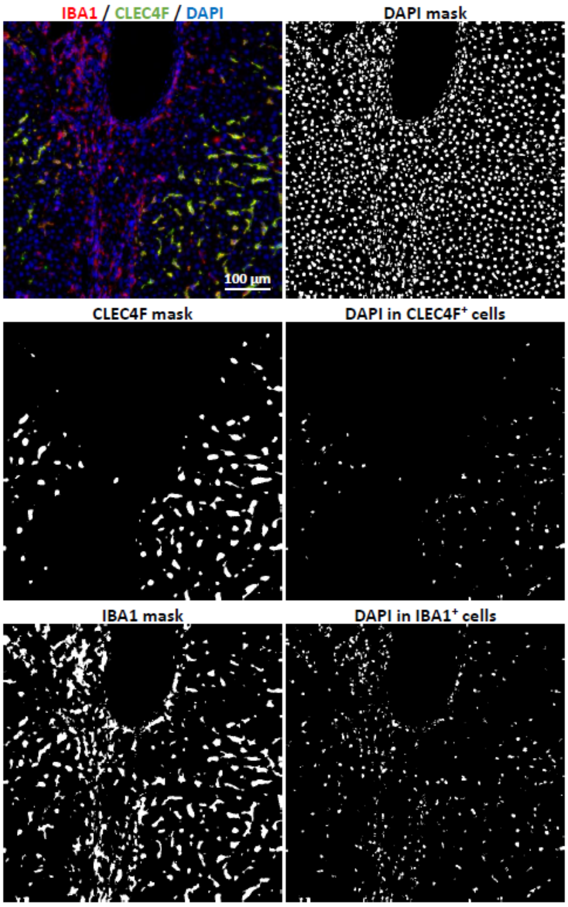

Figure S11d: Masks used for cell density map

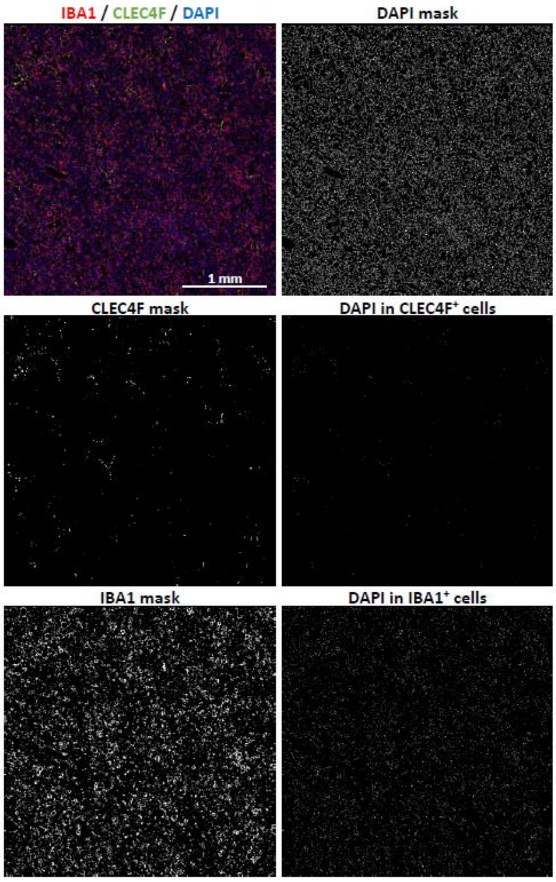

Figure S11e: Masks used for cell density map

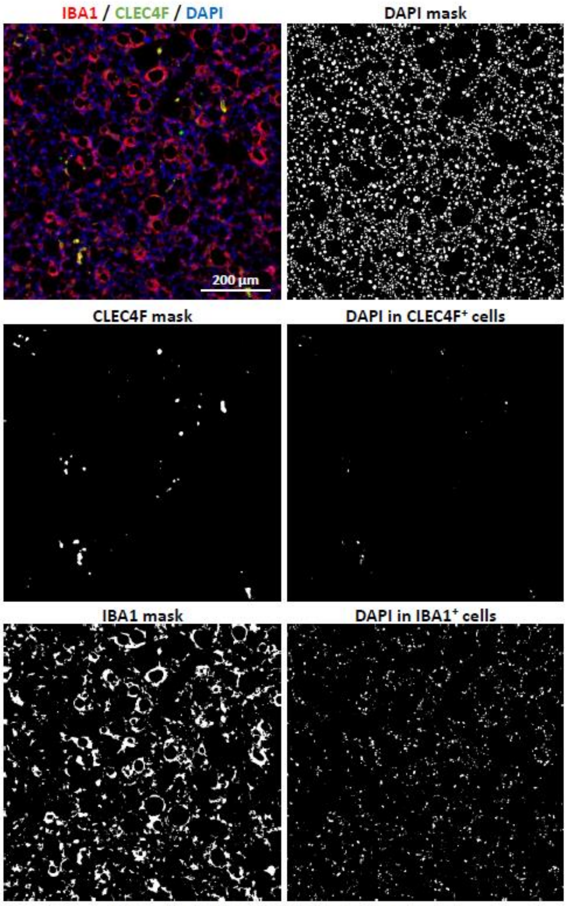

Figure S11f: Masks used for cell density map

Figure S11. Masks used for cell density map. Masks for DAPI, CLEC4F and IBA1 staining applied to generate the data depicted in Figure 5b–d were obtained using Ilastik. IBA1 and CLEC4F masks were further applied to the DAPI mask in order to select the corresponding nucleus attributable to IBA1⁺CLEC4F⁻ or IBA1⁺CLEC4F⁺ expressing cells. (**a**,**c**,**e**) Whole scanned areas and (**b**,**d**,**f**) enlarged areas.

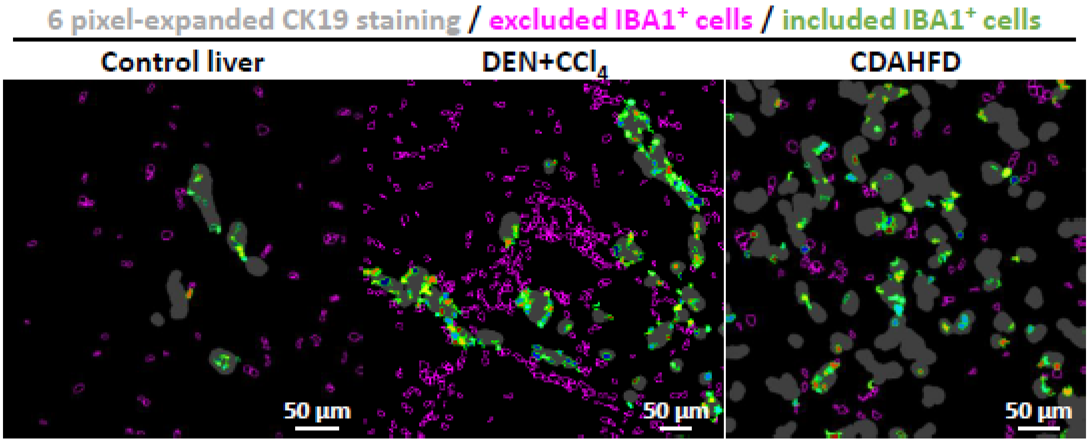

Figure 12. $CK19^+$ cell neighbor analysis. CellProfiler was used to quantify $IBA1^+$ cells in the $CK19^+$ cell neighborhood. A CK19-positive cell mask was generated using Ilastik, then expanded by 6 pixels in CellProfiler. This mask was applied on the $IBA1^+DAPI^+$ objects identified as indicated in Figure S11. The expanded CK19 mask is shown in grey, $IBA1^+$ $DAPI^+$ cells present within 6 pixels from $CK19^+$ cells are depicted in green, and excluded $IBA1^+DAPI^+$ cells in magenta.

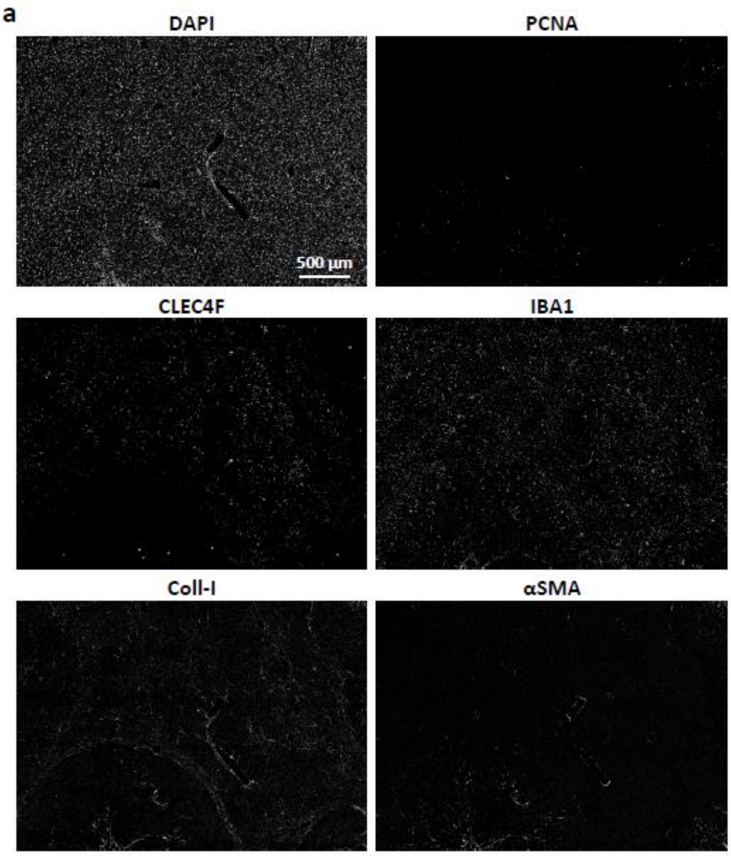

Figure S13a: Single channel pictures and masks used for tumor region analysis

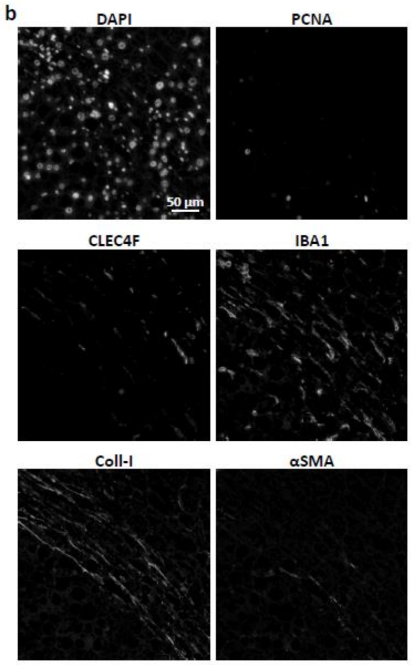

Figure S13b: Single channel pictures and masks used for tumor region analysis

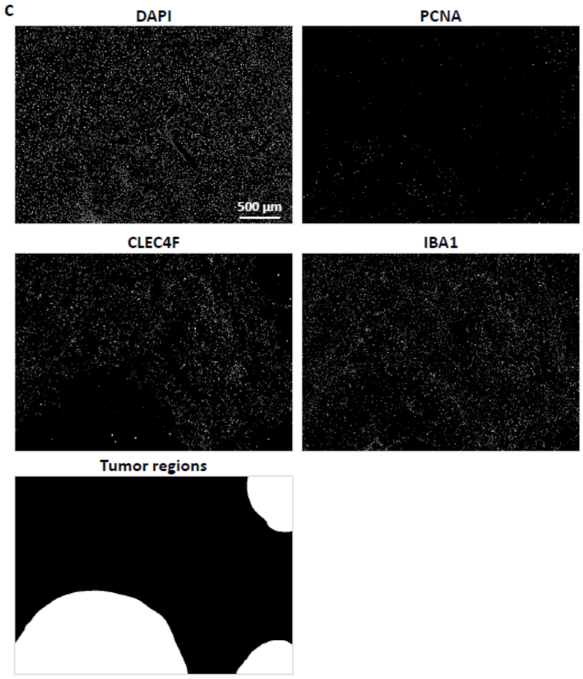

Figure S13c: Single channel pictures and masks used for tumor region analysis

Figure S13. Single channel pictures and masks used for tumor region analysis. (**a**,**b**) Single channel pictures from Figure 6a. (**c**) Masks used for cell numbering and tumor region analysis.

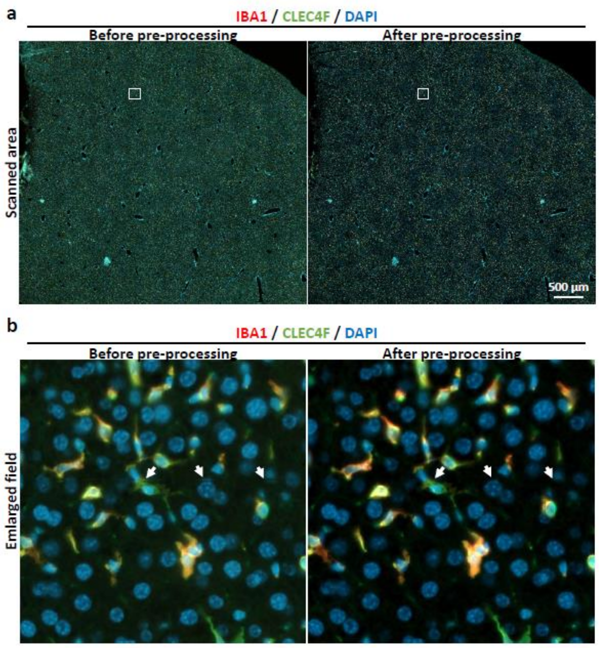

Figure S14: Stitching and background subtraction following large field scanning greatly enhance image quality

Figure S14. Stitching and background subtraction following large field scanning greatly enhance image quality. (**a**) Tile stitching and background subtraction were performed by using the ZEISS ZEN software on whole scanned areas after the acquisition of DAPI (blue), IBA1 (red) and CLEC4F (green) staining. (**b**) Enlarged area from the upper panel. White arrows indicate noticeable changes to the original pictures.

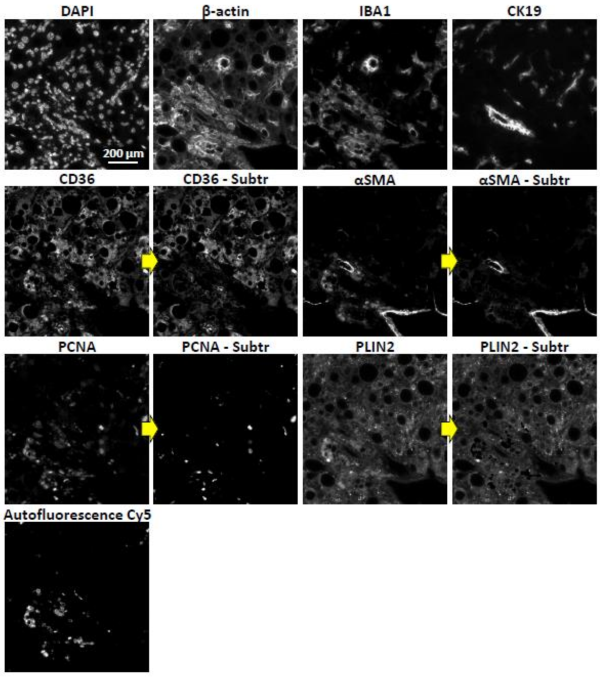

Figure S15a: Autofluorescence subtraction may enhance image quality

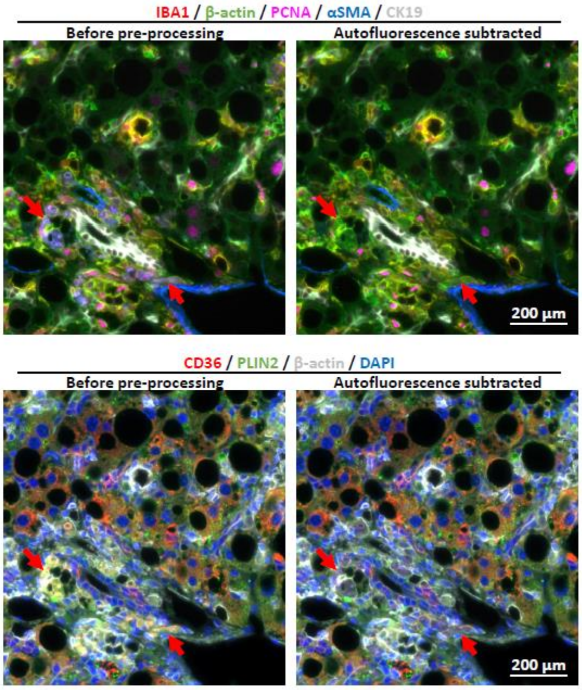

Figure S15b: Autofluorescence subtraction may enhance image quality

Figure S15. Autofluorescence subtraction may enhance image quality. (**a**) Single immunostaining pictures a CDAHFD fed mice, are depicted in grayscale. The autofluorescence acquired by using the Cy5 filter has been subtracted from the CD36, αSMA, PCNA and PLIN2 images as indicated. (**b**) Color merges reveal the revelance of rigorous autofluorescence subtraction. Red arrows show areas in which the autofluorescence subtraction may alter the conclusions drawn from these immunostainings.

Table S1: Antibodies used in this study.

	Abbreviation Used in This Report	Antigen Full Name	Manufacturer and Catalog #	Clone #	Host Species	Antibody Dilution in PBS+1%BSA
**Primary Antibodies**	αSMA	Alpha-smooth muscle actin	Agilent #M085129-2	1A4	Mouse	1:400
	β-actin	Beta-actin	Santa Cruz #sc-47778	C4	Mouse	1:200
	CD4	T-cell surface glycoprotein CD4	ThermoFisher #14-9766-82	4SM95	Rat	1:200
	CD8	T-cell surface glycoprotein CD8 alpha chain	ThermoFisher #14-0808-82	4SM15	Rat	1:200
	CD11b	Integrin alpha-M/beta-2	Abcam #ab133357	EPR1344	Rabbit	1:1,000
	CD16	Low affinity immunoglobulin gamma Fc region receptor III-A	Abcam #ab183354	SP175	Rabbit	1:500
	CD36	Adipocyte membrane protein	Abcam #ab133625	EPR6573	Rabbit	1:200
	CD45R	Receptor-type tyrosine-protein phosphatase C	Abcam #64100	RA3-6B2	Rat	1:200
	CD163	Scavenger receptor cysteine-rich type 1 protein M130	Leica #CD163-L-CE	10D6	Mouse	1:500
	CLEC4F	C-type lectin domain; family 4, member F	R&D Systems #MAB2784	370901	Rat	1:1,000
	CK19	Cytokeratin 19	TROMA-III was deposited to the DSHB by Kemler, R. (DSHB Hybridoma Product TROMA-III)	TROMA-III	Rat	1:200
	Coll-I	Collagen-I (mouse)	Abcam #ab21286	polyclonal	Rabbit	1:200
	Coll-I	Collagen I (human)	Abcam #ab34710	polyclonal	Rabbit	1:200
	Desmin	Desmin	Abcam #ab15200	polyclonal	Rabbit	1:200
	IL-17	Interleukin-17A	R&D Systems #AF-317-NA	polyclonal	Goat	1:200
	IBA1	Ionized calcium binding adaptor molecule 1	EMD Millipore #MABN92	20A12.1	Mouse	1:1,000
	IBA1	Ionized calcium binding adaptor molecule 1	VWR #100369-764	polyclonal	Rabbit	1:1,000
	Na:K ATPase	Anti-alpha 1 Sodium Potassium ATPase	Abcam #ab7671	464.6	Mouse	1:200
	PCNA	Proliferating cell nuclear antigen	Abcam #ab29	PC10	Mouse	1:10,000
	panCK	Wide spectrum Cytokeratin	Abcam #ab9377	polyclonal	Rabbit	1:500
	PD-1	Programmed cell death protein 1	R&D Systems #AF1021	polyclonal	Goat	1:200
	PLIN2	Perilipin-2	Abcam #ab52356	polyclonal	Rabbit	1:200
	S100a9	S100 calcium binding protein A9	Abcam #ab63818	polyclonal	Rabbit	1:5,000
	**Target Species**	**Fluorochromes**	**Manufacturer and Catalog #**	**Clone #**	**Host Species**	**Antibody Dilution in PBS+1%BSA**
**Secondary Antibodies**	Mouse IgG	Alexa Fluor® 488	Cell Signaling #4408S	polyclonal	Goat	1:500
		Alexa Fluor® 647	Cell Signaling #4410S	polyclonal	Goat	1:500
		Amplification kit, DyLight® 488	Vector #DK-2488	polyclonal	Goat/Horse	RTU
	Rabbit IgG	Alexa Fluor® 488	Cell Signaling #4412S	polyclonal	Goat	1:500
		Alexa Fluor® 555	Cell Signaling #4413S	polyclonal	Goat	1:500
		Alexa Fluor® 647	Cell Signaling #4414S	polyclonal	Goat	1:500
		Amplification kit, DyLight® 488	Vector #DK-1488	polyclonal	Goat/Horse	RTU
	Rat IgG	Alexa Fluor® 555	Cell Signaling #4417S	polyclonal	Goat	1:500
		Alexa Fluor® 647	Cell Signaling #4418S	polyclonal	Goat	1:500
	Goat IgG	CF™750	Merck #SAB4600444-125UL	polyclonal	Donkey	1:500

Abbreviations: BSA: Bovine serum albumin, CD: Cluster of differentiation, IgG: Immunoglobulin G, PBS: Phosphate-buffered saline, RTU: Ready-to-use.

Supplementary Reference

1. Eliceiri, K.W.; Michael R.B.; Ilya, G.G.; Luis, I.; Manjunath,B.S.; Maryann, E.M.; Robert, F.M.;Hanchuan, P.;Anne, L.P.;Badrinath, R.; et al. Biological imaging software tools. *Nat. Methods.* **2012**, *9*, 697–710.
2. Guiet, R.; Burri, O.; Seitz, A. in *Computer Optimized Microscopy: Methods and Protocols* (eds Elena Rebollo & Manel Bosch) 23–37 (Springer New York, 2019).
3. Corrigan, A.M.; Karlsson,J.; Wildenhain, J.; Knerr, L.; Halvarsson, M.O.; Karlsson, M.; Lünse, S.; Wang, Y. IA-Lab: A MATLAB framework for efficient microscopy image analysis development, applied to quantifying intracellular transport of internalized peptide-drug conjugate *PLoS One*. **2019**, *14*, e0220627, doi:10.1371/journal.pone.0220627.
4. Lopes, A.; Billard, E.; Casse, A.; Villéger, R.; Veziant, J.; Roche, G.; Carrier, G.; Sauvanet, P.;Briat, A.; Pagès, F. Deciphering the immune microenvironment of a tissue by digital imaging and cognition network *Sci Rep*. **2018**, *8*, 16692, doi:10.1038/s41598-018-34731-x.
5. Parra, E. R.; Francisco-Cruz, A.; Wistuba, I.I. State-of-the-Art of Profiling Immune Contexture in the Era of Multiplexed Staining and Digital Analysis to Study Paraffin Tumor Tissues *Cancers (Basel)*. **2019**, *11*, 247, doi:10.3390/cancers11020247.

© 2020 by the authors. Licensee MDPI, Basel, Switzerland. This article is an open access article distributed under the terms and conditions of the Creative Commons Attribution (CC BY) license (http://creativecommons.org/licenses/by/4.0/).
